# Dietary Compounds Influencing the Sensorial, Volatile and Phytochemical Properties of Bovine Milk

**DOI:** 10.3390/molecules25010026

**Published:** 2019-12-19

**Authors:** Holly J. Clarke, Carol Griffin, Dilip K. Rai, Tom F. O’Callaghan, Maurice G. O’Sullivan, Joseph P. Kerry, Kieran N. Kilcawley

**Affiliations:** 1 Food Quality and Sensory Science, Teagasc Food Research Centre, Moorepark, P61 C996 Fermoy, Ireland; Holly.clarke@teagasc.ie; 2 Sensory Group, School of Food and Nutritional Sciences, University College Cork, T12 R229 Cork, Ireland; maurice.osullivan@ucc.ie; 3 Food Industry Development, Teagasc Food Research Centre, Ashtown, D15 DY05 Dublin 15, Ireland; carol.griffin@teagasc.ie; 4 Food Biosciences, Teagasc Food Research Centre, Ashtown, D15 DY05 Dublin 15, Ireland; dilip.rai@teagasc.ie; 5 Food Chemistry and Technology, Teagasc Food Research Centre, Moorepark, P61 C996 Fermoy, Ireland; tom.ocallaghan@teagasc.ie; 6 VistaMilk, SFI Research Centre, Moorepark, Fermoy, P61 C996 Co. Cork, Ireland; 7 Food Packaging Group, School of Food and Nutritional Sciences, University College Cork, T12 R229 Cork, Ireland; joe.kerry@ucc.ie

**Keywords:** dairy, feeding system, volatile organic compounds (VOCs), sensory, isoflavones

## Abstract

The main aim of this study was to evaluate the volatile profile, sensory perception, and phytochemical content of bovine milk produced from cows fed on three distinct feeding systems, namely grass (GRS), grass/clover (CLV), and total mixed ration (TMR). Previous studies have identified that feed type can influence the sensory perception of milk directly via the transfer of volatile aromatic compounds, or indirectly by the transfer of non-volatile substrates that act as precursors for volatile compounds. In the present study, significant differences were observed in the phytochemical profile of the different feed and milk samples. The isoflavone formonoetin was significantly higher in CLV feed samples, but higher in raw GRS milk, while other smaller isoflavones, such as daidzein, genistein, and apigenin were highly correlated to raw CLV milk. This suggests that changes in isoflavone content and concentration in milk relate to diet, but also to metabolism in the rumen. This study also found unique potential volatile biomarkers in milk (dimethyl sulfone) related to feeding systems, or significant differences in the concentration of others (toluene, p-cresol, ethyl and methyl esters) based on feeding systems. TMR milk scored significantly higher for hay-like flavor and white color, while GRS and CLV milk scored significantly higher for a creamy color. Milk samples were easily distinguishable by their volatile profile based on feeding system, storage time, and pasteurization.

## 1. Introduction

The effect of bovine diet on the composition and flavor profile of milk is well documented [1,2,3]. However, conflicting results exist on the effect of feeding systems on the flavor and abundance of volatile organic compounds (VOCs) in dairy products, and their impact on the sensory perception of milk. Studies suggest that certain VOCs in milk could prove to be useful metabolic markers in tracing animal diets [4,5]. Alterations to feeding systems have been shown to effect milk fat composition, protein content, urea, citrate, and soluble calcium (list not exhaustive), which can subsequently influence the oxidative stability and flavor of the milk [6]. The review by Chilliard, et al. [7] summarized the effects of forage type on milk fat and composition, and highlighted the need to evaluate the impact of feeding systems on other aspects of milk fat quality, such as flavor and oxidative stability. Milk produced from many supplemented and altered diets have been investigated, including supplementation with flaxseed [8], lipid complex [9], crude protein [10], iodine [11] marine algae [12], oregano and caraway essential oils [13], hull-less barley [14] and sunflower/fish oil [15]. These studies focused mainly on animal production performance, milk composition, milk yield, milk fatty acid composition, and to a lesser extent on the flavor and sensory characteristics of milk. The study by O’Callaghan, et al. [16] investigated the influence of four supplemental feed choices for pasture-based cows on the fatty acid and volatile profile of milk. Some studies have also evaluated the effect of storage conditions on the microbiological quality of milk [17,18]. In the present study, the volatile profile and free fatty acid (FFA) content of the milk samples were evaluated over a 14-day storage period at 4 °C in order to ascertain the level of lipid oxidation occurring within the milk, and to track volatile compounds forming or changing during refrigerated storage. Free fatty acids (FFAs) in milk are produced by two mechanisms, namely incomplete esterification in the mammary gland before lipid excretion [19] or lipid hydrolysis after milking and during storage [20]. The FFAs influence product quality, flavor, nutrition, and texture, and thus accurate quantification is important for quality control as well as research and development purposes [21]. FFA levels > 1.5 mmol/L are unacceptable to most consumers [22]. A number of factors including individual animals, feeding system, stage of lactation, farm practices, bacterial contamination, and storage quality influence the level of FFA in milk [23]. Increased levels of unsaturated fatty acids bound in the lipid molecules (triacylglycerol or phospholipids) or as FFA have been shown to increase the susceptibility of milk to lipid oxidation [24], thus impacting negatively on quality and sensory properties. Increased levels of short and medium chain FFA in particular have been shown to be responsible for off-flavors described as rancid, butyric, and astringent [25]. Increased levels of ethyl esters of short-chain fatty acids, particularly ethyl butanoate and ethyl hexanoate, impart a fruity off-flavor in milk [26].

Furthermore, four important isoflavones (apigenin, daidzein, formononetin, and genistein) with potentially important sensory implications were also investigated. Isoflavones are a group of phytoestrogens with estrogenic or hormone-like properties, and are known to have positive effects on various diseases, including atherosclerosis, osteoporosis, and some cancers [27], but may also act as substrates for biomarkers of pasture feeding and influence sensory properties through the degradation of odor active compounds [1,5]. Isoflavones in bovine milk are likely present as a result of the direct transfer from feeds including leguminous plants such as clover and soybean, which are naturally rich in phytoestrogens. Dairy produce from pasture-based farming systems is considered more natural by consumers from an animal welfare and environmental standpoint [28]. Feeding total mixed ration (TMR) and housing cows indoors year-round is a widely implemented farming practice in the United States and many parts of Europe. Such systems have been linked with increased lameness, reduced comfort, and increases in mastitis, all of which affect animal performance [29,30]. Therefore, the main aim of this study was to investigate the effect of three widely implemented feeding regimes, namely outdoors on perennial ryegrass (*Lolium perenne* L.), outdoors on perennial ryegrass/white clover (*Trifolium repens* L.), and indoors on TMR on the phytochemical, volatile, and descriptive sensory profiles of bovine milk. To the authors’ best knowledge, no published study has investigated the impact of feeding systems on the phytochemical, volatile, and descriptive sensory profiles of bovine milk. 

## 2. Results and Discussion

### 2.1. Microbial Analyses

Each raw and pasteurized milk sample was tested for the presence of coliforms and enterococci in addition to the total bacteria count. Results are presented in Supplementary Table S1. As expected, there was a significant decrease in microbial activity post pasteurization and no coliforms were detected.

### 2.2. Pasteurized Milk Compositions

The fat, protein, lactose, true protein, and casein contents for the milk samples taken at mid and late lactation are available in Supplementary Table S2. Significant differences were observed between the levels of fat, protein, lactose, true protein, and casein at *p* = 0.001 based on the stage of lactation, in agreement with the study by O’Callaghan, et al. [31] who reported significant differences between fat, protein, and casein but not lactose over an entire lactation.

### 2.3. Free Fatty Acid Analyses

Results showed a significant increase in C18:1 in grass (GRS) milk samples from day three to 14. In the grass/clover (CLV) samples, significant differences were observed across all the 11 FFAs and finally, significant increases were observed in the levels of C6, C8, C14, C16, C18 and C18:1 in the TMR samples from day three to 14 (Tables S3 and S4). Variations in the FFA content of milk in the present study are in agreement with the study conducted by Villeneuve, et al. [32] whereby levels of FFA with a chain length of four (butanoic acid) to 16 (tetradecanoic acid) were found to be higher in milk from cows fed pasture than milk from cows fed silage produced from timothy grass swards. Similarly, levels of C18:1 were higher in milk from pasture compared with milk from silage (TMR day nine milk was omitted from results processing due to possible microbial contamination). The levels of FFA across all samples, particularly the short chain FFA, were low and so were unlikely to cause any objectionable off-flavors associated with FFA described above. This is also indicative of good quality milk.

### 2.4. Phytochemical Analyses

**Figure 1:**
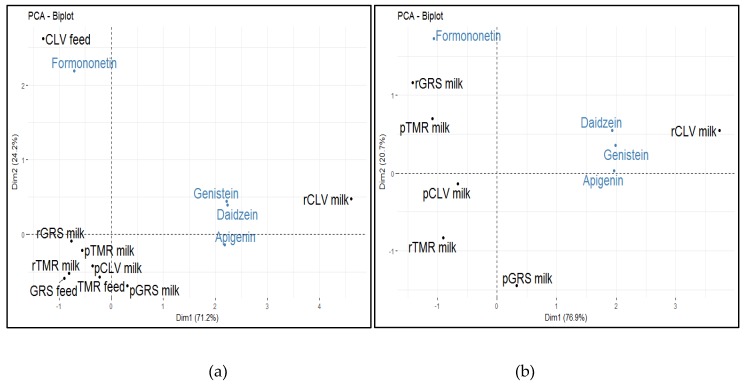
(**a**) Principal component analysis (PCA) biplot showing the correlations between the isoflavones (apigenin, daidzein, formononetin, and genistein) identified in feed samples (grass, grass/clover and TMR) samples and the corresponding raw (r) and pasteurized (p) (grass (GRS), clover (CLV) and total mixed ration (TMR) milk samples as determined by liquid chromatography tandem mass spectrometry (LC-MSMS); (**b**) PCA biplot showing the correlation of the isoflavones (apigenin, daidzein, formononetin, and genistein) to raw (r) and pasteurized (p) GRS, CLV and TMR milk samples as determined by LC-MSMS.

Isoflavones are important as they have the ability to be directly transferred from feed to milk, and subsequently to be reduced to compounds that can potentially impact the sensory properties of milk and other dairy products. In particular, formononetin has been linked to the production of p-cresol [33]. At day three, p-Cresol was not detected in the milk samples, but was detected in all samples at days nine and 14 of storage. It is possible that it was present in the milk samples at day three in sulfonated form or below levels of detection, and was subsequently released by enzymatic action, specifically by arylsulfatase during the storage period [34]. The concentrations of formononetin were found to be significantly correlated to white clover (CLV) feed samples (Figure 1a). Levels of apigenin, daidzein, and genistein were found to be significantly different between the raw (r) and pasteurized (p) GRS, CLV, and TMR milk samples. Daidzein and genistein were highly correlated to rCLV milk and formononetin was more closely correlated with rGRS milk (Figure 1b). 

The concentration of formononetin was found to be highest in rGRS milk, as previously mentioned, and formononetin is likely degraded to p-cresol, a compound that has been associated with barnyard aroma in dairy products. Both r and p GRS milk had the highest levels of p-cresol at days nine and 14. Further, pGRS milk was also more correlated with barnyard aroma than the pCLV and pTMR. The significant correlation of formononetin to CLV feed samples (Figure 1) is expected as leguminous plants such as clover are naturally rich in phytoestrogens [35]. The difference in levels between r and p milks suggests an effect of pasteurization on the compounds, but it is possible that some or all of the formononetin present in the samples was demethylated to daidzein, which is highest in rCLV milk and not detected in the corresponding CLV feed samples (Figure S1). It is also possible that daidzein was further reduced via hydrogenation and ring scission to equol (a microbial metabolite of isoflavone with high estrogenic activity) [36] or metabolized to O-desmethylangolensin [37]. The composition of the individual bovine gut microflora impacts largely on the metabolism of daidzein and subsequently on the rate of equol excretion [35]. Although formononetin is closely correlated to rGRS milk, the TMR feeding system implemented in this study is partly soya-based, in addition to containing grass silage and maize silage, which could explain its proximity to pTMR milk (Figure 1). The other three isoflavones (daidzein, genistein, and apigenin) are significantly correlated to rCLV milk. Numerous isoflavones are readily reduced or converted to other phytoestrogens. As previously mentioned, formononetin can be converted to other isoflavones such as daidzein and subsequently equol [38]. Genistein and daidzein both require degradation of the active compound by gut microflora in order to become bioavailable. Note that S(–)-equol is the active metabolite of daidzein. Genistein, a metabolite of biochanin A, is generally metabolized to glucuronides and sulfate conjugates [39]. Genistein can also be degraded to the higher homolog of p-cresol, 4-ethyl-phenol, by gut microflora [40]. It is known thay 4-Ethyl-phenol is an inactive metabolite with no estrogenic activity. Further, p-Cresol-sulfate has been shown to be a gut-mediated metabolite of genistein. Apigenin has been reported to be metabolized to luteolin, mediated by the enzyme cytochrome P450 [41]. Isoflavone metabolites are also known to be excreted in the urine of ruminants [42] and so losses occur. Moreover, Turner [43] has postulated that the epithelial cells in the mammary gland may only be semi-permeable to estrogenic compounds resulting in a limited transfer from the blood to the milk. Thus, it seems likely that any isoflavones that are present through ingestion can be metabolized to odor-active compounds that potentially impact the sensory properties of bovine milk.

### 2.5. Volatile Analyses (Feed, Raw and Pasteurized Milk)

Volatile profile analysis by headspace solid-phase microextraction gas-chromatography mass spectrometry (HS-SPME GCMS) was performed on the GRS, CLV, and TMR feed samples, and on the r and p GRS, CLV, and TMR milk samples on days 3, 9, and 14 of refrigerated storage. The transfer of VOCs from feed to bovine milk is well documented, and studies have shown that volatile compounds in forage and feed can enter milk by two mechanisms, namely absorption via the digestive tract (rumen or intestine) before diffusing into the blood and subsequently the mammary gland, and/or through the pulmonary system wherein volatiles present in the air are inhaled, absorbed through the lungs, enter the blood steam, and diffuse into the mammary gland [1,44]. Respectively, 90 and 104 compounds were identified in GRS and CLV feed samples, consisting mainly of aldehydes, ketones, esters, alcohols, and hydrocarbons. A further 94 compounds were identified in TMR samples consisting mainly of aldehydes, ketones, esters, alcohols, acids, and hydrocarbons (Supplementary Figures S2 and S3).

Eleven aldehydes, 10 ketones, 30 esters, 10 alcohols, seven acids, two fatty acid esters, one terpene, four furans, five hydrocarbons, two phenols, two sulphur compounds, two lactones, four pyrazines, and one ether compound varied significantly (*p* < 0.001) between feed types (Table 1). As well as the direct transfer from feed, alterations in VOCs in milk can occur during pasteurization (thermal) or storage (oxidative, microbial and enzymatic). Of the 32 volatile compounds identified in the feed samples and the corresponding raw milk samples, 20 were identified across all samples (decanal, heptanal, hexanal, nonanal, 2-heptanone, 2-pentanone, acetone, acetophenone, 2-methyl-1-butanol, 3-methyl-1-butanol, cumene, mesitylene, 2,4-dimethylfuran, 1,3-bis(1,1-dimethylethyl)-benzene, 2,4-dimethyl-benzaldehyde, p-xylene, tert-butylbenzene, toluene, dimethyl sulphide and vinylisopentyl ether). It is probable that some of these compounds were transferred directly from the feed to the milk. Decanal (sweet aldehydic), heptanal (green, fatty, herbal), hexanal (green, fatty), nonanal (waxy, orange-peel, fatty), octanal (aldehydic, waxy, fatty), and pentanal (fermented, cardboard-like, bready, nutty) are lipid oxidation products resulting from fatty acid degradation. Further, [32,45] 2-heptanone (fruity, spicy, sweet) and 2-pentanone (sweet, fruity, ethereal) are secondary oxidation products. Acetone (hay, earthy, wood pulp) has previously been reported to originate from the diet of cows [19], while acetophenone (floral) is a product of phenylalanine metabolism [46] and is also a product of the Maillard reaction, which has been attributed to the heated or sterilized flavor of ultra-heat treated milk [47]. Moreover, 2-Methyl-1-butanol (roasted, wine, onion, fruity) and 3-methyl-1-butanol (fermented) may have originated from the degradation of isoleucine and leucine, respectively, by *Saccharomyces cerevisiae*, as well as other yeasts [48]. They may have also been produced from their corresponding methylketones by reductase activity or by lactic acid bacteria [49]. Further, 1-Pentanol (fermented, bready, yeasty) is derived from the primary aldehyde pentanal by oxidation [1] and α-Pinene (herbal) is most likely derived from plant diet, and was highest in GRS feed, rGRS, and pGRS milk samples. Cumene (gasoline like), also a plant derived alkylbenzene [50], was highest in rCLV and pCLV milk samples. Mesitylene (sweet) is a benzene derivative that is structurally related to toluene (nutty, bitter, almond, plastic), m-xylene (plastic) and ethylbenzene (gasoline-like), and was found to be highest in CLV feed samples. It is possible that mesitylene is formed through carotenoid degradation, which is observed for other benzene compounds, but it has also been found unchanged in the blood and urine of human patients as a result of air exposure, and so could be introduced through the inhalation pathway [51]. It is possible that the furan 2,4-dimethylfuran (odor unknown) in the milk samples is due to the thermal degradation of certain amino acids, including serine and cysteine [52]. Further, 1,3-Bis(1,1-dimethylethyl)-benzene (odor unknown), 2,4-dimethyl-benzaldehyde (naphthyl), and tert-butylbenzene (phenolic) could have entered the milk through inhalation or ingestion, and were possibly partially degraded to phenol (phenolic) [53], however, some benzene compounds are thought to be products of the Strecker reaction [54]. For example, p-Xylene (sweet) may be present as a result of β-carotene degradation in the rumen [55], or from direct transfer from feed [56]. Toluene, a product of β-carotene degradation [1], is also derived from plant diet and is highest in CLV feed samples, but was higher in r and p GRS milk samples. Dimethyl sulphide (sulphurous, onion, cabbage) has been shown to be transferred from the rumen to milk [57] and two possible precursors. Their originating in plant materials may account for this, namely dimethyl-fl-propiothetin and methylmetbioninesulphonium salt [58]. Dimethylsulfoniopropionate can also undergo degradation via cleavage to dimethyl sulphide or demethylation and demethiolation to methanethiol [59], which was detected in all feed samples, but only in rCLV milk samples at day 14 of storage. Dimethyl sulfone (sulphurous, burnt) is also a product of methionine degradation and a product of plant diet [32,60]. Thus, the higher levels identified in GRS feed, and in the corresponding r and p GRS milk samples, possibly from the higher concentrations of digestible proteins, are in agreement with previous studies [1,61]. Further, 2-Hexanone (fruity, meaty, buttery), and methyl isobutyl ketone (green, fruity) are likely lipid oxidation products [1,62] and were identified in all milk samples. Acetyl valeryl (2,3-heptanedione) (buttery) was only identified in CLV feed samples. Concentrations of acetyl valeryl in cheese products has been previously associated with the presence of certain *Lactococcus Lactic* strains, milk storage temperatures before cheese making [63], and seasonal variations [64], suggesting that it could be dependent on feed composition. The levels of acetyl valeryl increased in r and p CLV milk samples during storage at 4 °C. Further, 2-Butanone (buttery, sour milk, ethereal) derived from carbohydrate metabolism, was only detected in TMR milk samples, and has previously been reported to originate from the diet of cows and from carbohydrate metabolism [19]. Ethylbenzene, likely a product of carotenoid degradation, and 1-hexanol, derived from the aldehyde hexanal [5] were also detected in rTMR milk only.

Many newly formed compounds were identified in milk samples at day 14 of storage, in particular esters. Moreover, the levels of certain compounds present on day three of analysis increased or decreased over storage, highlighting that storage time has an effect on the volatile profile of bovine milk (Table 2 and Supplementary Table S5). Rashid et al. [65] investigated the effect of storage time on the concentrations of volatiles known to cause off-flavors in milk. Results showed the ability of certain compounds to both increase and decrease over time at 4 and 7 °C. Any fluctuations occurring throughout storage are likely due to lipid hydrolysis [32], lipid oxidation [66], microbial changes by indigenous or bacterial lipases [49], or by enzymatic action [20].

Esters and aldehydes were more closely correlated with TMR milk samples. Esters of short chain fatty acids (C4–C10) are important aroma active compounds [26] that are responsible for fruity off-flavors in milk [67,68]. It is possible for esters to be formed through esterification reactions (the formation of esters from alcohols and carboxylic acids) or alcoholysis (the production of esters from alcohols and acylglycerols or from alcohols and fatty acyl-CoAs derived from metabolism of fatty acids, amino acids and/or carbohydrates) [26]. Esters in pasteurized milk are occasionally present as a result of post-pasteurization microbial contamination and microbial activity [69,70]. Ethyl butanoate (fruity) was identified in rGRS and rTMR samples at day 14 of storage and ethyl hexanoate (fruity, malty pineapple, waxy) was identified in r and p GRS milk at day nine and increased at day 14. It was also identified in rCLV and rTMR samples at day 14 only. Ethyl butanoate was identified in rGRS and rTMR milk at day 14 of storage but was not identified in any p milk samples. The contribution of esters to the flavor of milk is concentration-dependent, and at low levels, esters contribute positively to the overall flavor balance; but at high concentrations they can cause a fruity defect as mentioned previously [26]. Hydrocarbons and sulphur compounds were more closely associated with GRS and CLV milk samples. Al-Attabi et al. [71] reported that sulfur compounds such as hydrogen sulfide, methanthiol, dimethyl sulfide, and dimethyl trisulfide were present in commercial ultra-heat-treated milk samples at levels above their documented odor thresholds. The same study identified carbon disulfide, dimethyl sulfide, dimethyl sulfoxide, and dimethyl disulfide in pasteurized milk samples, although below their reported threshold values. Sulphur compounds are thought to be important contributors to the cooked flavor in milk.

**Figure 2:**
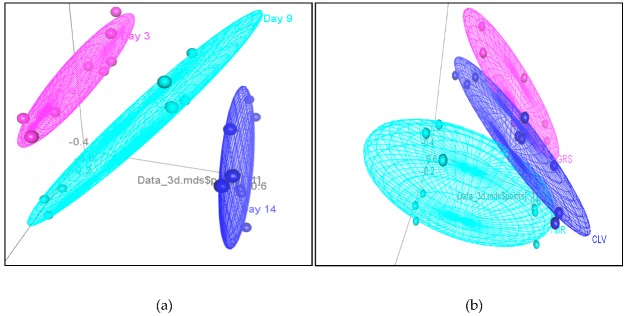

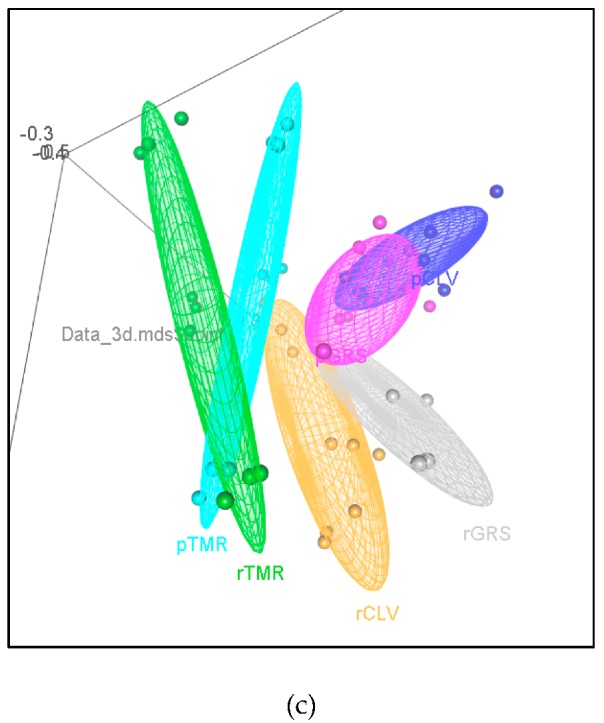
(**a**) 3D pl ot demonstrating the effect of storage time (days) on the volatile profile of the raw milk grass (rGRS), raw milk grass/clover (rCLV) and raw milk total mixed ration (rTMR) milk samples; pink—day three, light blue—day nine and dark blue—day 14; (**b**) 3D plot demonstrating the effect of feeding system (grass (GRS), grass/clover (CLV) and total mixed ration (TMR)) on the volatile profile of the rGRS, rCLV and rTMR milk samples; pink—GRS, light blue—TMR and dark blue—CLV; (**c**) 3D plot demonstrating the effect of pasteurization on the volatile profile of the r and p GRS, CLV and TMR milk samples. Grey—rGRS grass, pink—pGRS, orange—rCLV, dark blue—pCLV, light blue—pasteurized TMR and green—raw TMR. p = pasteurized, r = raw.

Significant differences were observed between the GRS, CLV, and TMR milk samples based on storage time, feeding system, and pasteurization (Figure 2a–c, respectively). Differences between the rGRS, rCLV, and rTMR milk samples at day three were dominated by alcohols (3) and aldehydes (2). Differences between the p milk samples based on feeding system at day three were dominated by aldehydes (3), alcohols (2) and hydrocarbons (2). Raw milk samples at day nine were dominated by aldehydes (6), alcohols (3) and ketones (3). Pasteurized milk samples at day nine were dominated by esters (6), ketones (6) and alcohols (4). r milk samples were significantly (*p* < 0.05) dominated by esters (9), ketones (7), alcohols (4), aldehydes (3) and hydrocarbons (4) after 14 days of storage while p milk samples contained higher levels of aldehydes (7), ketones (6), and hydrocarbons (5) at day 14. All results for the concentrations of volatile organic compounds identified in r and p milk samples are outlined in Table 2 and Supplementary Table S5, respectively.

**Figure 3:**
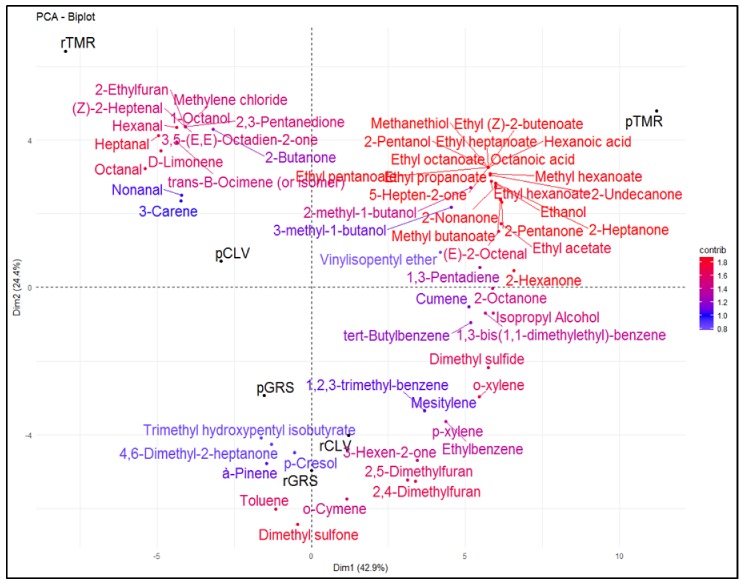
Principal component analysis (PCA) Biplot of raw (r) and pasteurized (p) grass (GRS), clover (CLV) and total mixed ration (TMR) milk samples and the top 60 volatile compounds contributing to the differences between the samples, identified by HS-SPME GCMS. Color gradient; low = white, mid = blue and high = red, midpoint set at 1.0.

Twenty eight compounds identified in the grass GRS feed samples were identified in the corresponding r milk samples (decanal, heptanal, hexanal, nonanal, octanal, pentanal, 2-heptanone, 2-hexanone, 2-pentanone, acetone, acetophenone, cyclohexanone, methyl isobutyl ketone, 2-methyl-1-butanol, 3-methyl-1-butanol, 1-pentanol, α-pinene, cumene, mesitylene, 2,4-Dimethylfuran, 2,4-Dimethyl-benzaldehyde, 1,3-Bis(1,1-dimethylethyl)-benzene, *p*-xylene, tert-Butylbenzene, toluene, dimethyl sulphide, dimethyl sulfone and vinylisopentyl ether). The same compounds were present in the CLV feed samples and the corresponding r milk samples excluding cyclohexanone, α-pinene, and methyl isobutyl ketone. Acetyl valeryl was the only compound present in CLV feed and corresponding r milks that was not in GRS samples. The majority of the same compounds were present in TMR feed and corresponding r milk, excluding octanal, pentanal, 2-hexanone, cyclohexanone, 1-pentanol, and dimethyl sulfone. The following three compounds, 2-butanone, 1-hexanol, and ethylbenzene, were identified in TMR feed samples, but not in GRS or CLV feed samples. Figure 3 demonstrates the correlation of the volatile compounds to the r and p milk samples.

Fifty-five compounds were identified in the p milk samples at day three, nine of which varied significantly. Further, 2-Methyl-1-butanol was significantly higher in pTMR samples, and has been linked to a malty, microbial-induced off-flavor related to the poor refrigeration of milk [19]. Further, 1-Pentanol (fermented, bready, yeasty, fusel) was significantly correlated with pCLV samples, and as previously mentioned, 1-pentanol is derived from pentanal [1], and its concentrations were linked to this aldehyde, which was also greater in CLV > GRS > TMR. Reportedly, 2-Butanone originates from the cows’ feeding system [19], specifically from carbohydrate metabolism which could explain why levels of this compound were highest in pTMR samples. The level of 3-Hexen-2-one (nutty, blue-cheese, plastic) was higher in GRS and CLV milk samples compared to TMR milk samples. It is likely that 3-hexen-2-one is derived from the aerobic oxidation of linoleic or linolenic acid (C18:2 and C18:3) [72]. Dimethyl sulfone was highest in the pGRS milk samples. Heptanal and nonanal were significantly correlated with r and p TMR milk samples and their presence has previously been reported in milk [73]. Both compounds are transferred from feed, but are also products of lipid oxidation [1]. Further, 3-Hydroxy-2,2,4-trimethylpentyl-ester-2-methyl-propanoic acid (sour, bitter, herb) was more closely correlated with GRS milk samples. It has been identified in numerous plant species [74,75,76] and as an odorant of some hardwood species [77]. In TMR milk, 2-Methyl-propanoic acid (Isobutyric acid) has previously been reported [1]. It has a characteristic sweet-like odor and is a plant metabolite produced from the intermediary hepatic and microbial metabolism of the amino acids valine and leucine [78,79]. However, conflicting results exist on whether 2-methyl-propanoic acid is transferred from feed to milk as Bingham, et al. [80] reported that no carryover was evident in the milk of cows supplemented with 170 mg/kg/day of the acid for 10 days due to the rapid metabolism of dairy cattle. It is also possible that the compound entered the milk through the inhalation pathway. Tert-butylbenzene is possibly derived from carotenoid degradation as observed with other benzene compounds and was highest in CLV milk samples. Toluene is a product of β-carotene degradation and has been identified as a potential biomarker for dairy products produced from pasture GRS > CLV > TMR [5], but is not very odor active [5]. Seventy-four compounds were identified in the p milk samples at day nine. Twenty-nine volatiles varied significantly. For example, (*E*)-2-octenal (fatty, green, cucumber) has previously been detected in milk fermented with *S. thermophiles* and was found to be an important contributor to the flavor of the milk [81]. Further, 2-Heptanone (cheesy, fruity, woody, herbal), 2-hexanone, 2-nonanone (fruity, sweet, green, earthy), 2-pentanone (fruity, wine, banana, ethereal), 2-undecanone (fruity, waxy, creamy, floral), and 5-hepten-2-one (citrus, green, apple lemongrass) are all ketone compounds commonly identified in milk, and some have been identified as thermally derived off-flavors linked to the level of fat in the milk [60]. All ketone compounds were highest in pTMR samples at day nine. Cumene is derived from benzene and its abundance was similar to that of benzene, in the order of TMR > GRS > CLV. Cumene has previously been identified in grass and plant material [50] and thus could be transferred directly from the feed. Dimethyl sulphide was highest in TMR samples and dimethyl sulfone was higher in GRS and CLV samples possibly due to the presence of more digestible proteins [1]. The ester compounds, ethyl (*Z*)-2-butenoate (fermented, chemical, caramel), ethyl acetate (ethereal, fruity, sweet), ethyl hexanoate, ethyl octanoate (wax, sweet, apple), ethyl pentanoate (fruity, acidic, green), methyl butanoate (fruity, apple, fusel), and methyl hexanoate (fruity, pineapple, ether), were all significantly higher in pTMR samples, possibly due to the amount of ethanol available to form ethyl esters, and methanol to form methyl esters, a reaction that can occur spontaneously, or be catalyzed by esterases or lipases produced by lactic acid bacteria [82,83]. Heptanal was more closely correlated with pGRS milk samples at day nine. Hexanal, a primary product of lipid oxidation (oleic and linoleic acid) is a well-known contributor to off-flavors in dairy products [84,85] and was found to be higher in pGRS milk at day nine. Nonanal and octanal were significantly correlated with pGRS samples at day nine and both compounds have previously been identified as thermally derived off-flavors in milk [60] and products of light-induced oxidation [73]. Pentanal was greatest in pCLV samples. Methanethiol (sulfurous, cabbage, garlic), is derived from the Strecker degradation of methinonine and also from riboflavin [71], and was only detected in pTMR samples. Methanethiol can also be easily oxidized to form dimethyl disulfide [86]. Styrene (balsamic, woody) is produced from the degradation of cinnamic acid and as a by-product of fungal and microbial metabolism [87,88], and was only detected in pGRS samples. Toluene concentrations were higher in GRS samples followed by CLV then TMR. Seventy eight compounds were identified in the milk samples at day 14, 15 of which varied significantly between the milk types: (*Z*)-2-heptenal (green, fatty); 1-octanol (waxy, green, mushroom) is a fatty alcohol that could be derived from octanal; 1-Pentanol increased in pGRS samples at day 14; 2-Butanone remained correlated with pTMR samples at day 14; 3,5-(*E*,*E*)-Octadien-2-one (grassy, fruity, green) is a product of linolenic acid degradation [89] and was significantly higher in pTMR samples, which may explain the perceived hay-like flavor in the pTMR samples; 4-Methyl-3-penten-2-one (honey, vegetable, earthy) was identified in pGRS and pCLV samples only, and was found to be more closely correlated with the pCLV samples; acetone, as previously mentioned, is thought to originate from the diet of cows [19] and was also higher in the pTMR sample; butanal (chocolate, pungent, musty), a primary aldehyde product of lipid oxidation [90] was detected in the pGRS and pTMR samples only, being most abundant in pTMR samples; heptanal, hexanal, nonanal and octanal were all more closely correlated with TMR samples at day 14, as these are all products of lipid oxidation [90] their increased concentrations in pTMR samples could indicate quality deterioration and an increase in off-flavors; pentanal remained correlated with pCLV samples after 14 days of storage; p-cresol (barnyard, cowy, phenolic) is derived from the metabolism of β-carotene and aromatic amino acids (mainly tyrosine) in the rumen and may be a potential biomarker for dairy products derived from pasture [5,91,92]. Further, p-Cresol was strongly correlated with pGRS milk. Tyrosine has been shown to be a precursor for the production of both p-cresol and phenol [93], and both compounds follow the same trend across all p milk samples with the exception of TMR samples at day nine. Additionally, p-Cresol may also be present from the metabolism of isoflavones in the feed [33]. Toluene was also found to be significantly correlated with the pGRS samples. In addition to the number of compounds increasing in the p milk samples throughout the storage period, the levels of numerous VOCs decreased, possibly due to the degradation and/or formation of secondary compounds. It is well known that pasteurization has an effect on certain volatile compounds in milk and can lead to losses or changes [1]. This can be seen as some compounds that are present in the r milk samples are absent in the corresponding p milk samples or vice versa. This is very evident for esters which could have been formed by heat-catalyzed esterification reactions [1,60], some aldehydes (possibly from the activation of lipid oxidation after heat treatment, autooxidation or light induced oxidation) and some ketones [46]. It has been noted that enzymatic and metabolic reactions that occur in raw milk during storage may also lead to the loss of compounds post pasteurization [46,94]. Storage time was also shown to have an effect on the volatile profile and this could be due to enzymatic reactions from microbes.

### 2.6. Sensory Analyses of Pasteurized Milk Samples 

**Figure 4:**
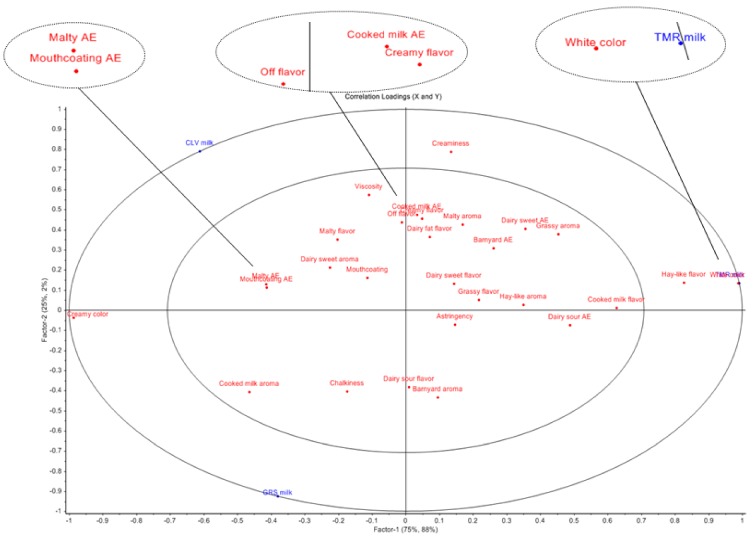
Multivariate data analysis partial least squares (PLS) regression plot of sensory descriptors for pasteurized milk samples; grass (GRS), grass/clover (CLV) and total mixed ration (TMR). AE denotes after effect. *p* = 0.05%.

**Figure 5:**
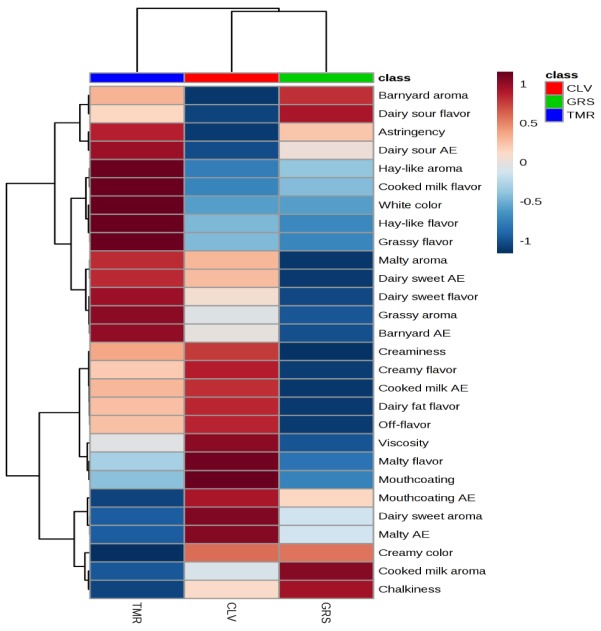
Hierarchal clustering analysis (Heatmap) of the average values for each sensory descriptor applied to the 3 pasteurized milk samples (grass (GRS), clover (CLV) and total mixed ration (TMR)) as determined by full descriptive sensory analysis (*n* = 7). Positive and negative correlations between diet treatment and sensory descriptors is denoted by +1 (red) and −1 (blue). AE: aftereffect.

It can be observed from Figure 4 and Figure 5 that there is considerable discrimination between the three milk samples. Three significant differences (*p* < 0.05) were observed between the three milk types; creaminess, color and hay-like flavor. A post hoc Tukey’s test showed that the difference in creaminess exists between GRS and CLV milk, the difference in color exists between TMR and the other two milks, with TMR milk scoring highest for white color and GRS and CLV milks scoring highest for creamy color. The significant difference in creaminess is likely to be linked to the higher level of fat in the CLV milk, as creaminess is linked to milk fat globules in dairy products [95]. Fat is in the form of emulsified globules in liquid dairy products which are perceived as smooth and creamy [96]. The fatty acid profile of milk also has an impact on texture, the ratio of oleic acid (C18:1; low melting point) to palmitic acid (C16:0; high melting point) has been used as a measure of hardness in cheese and butter [97]. Faulkner, et al. [1] reported that milk samples produced from pasture scored significantly higher for viscosity, possibly due to the lower ratio of oleic acid to palmitic acid. In this study, CLV samples scored significantly higher for creaminess and contained a lower ratio of oleic acid to palmitic acid followed by GRS and TMR samples, which is in agreement with the previous studies [97]. Free FA profile also impacts the surface tension and foaming capacity of milk, which contribute to texture [21]. β-Carotene content is responsible for the difference in color with the study by Martin, et al. [97] who concluded that dairy products produced from cows fed pasture have a higher yellow intensity.

**Figure 6:**
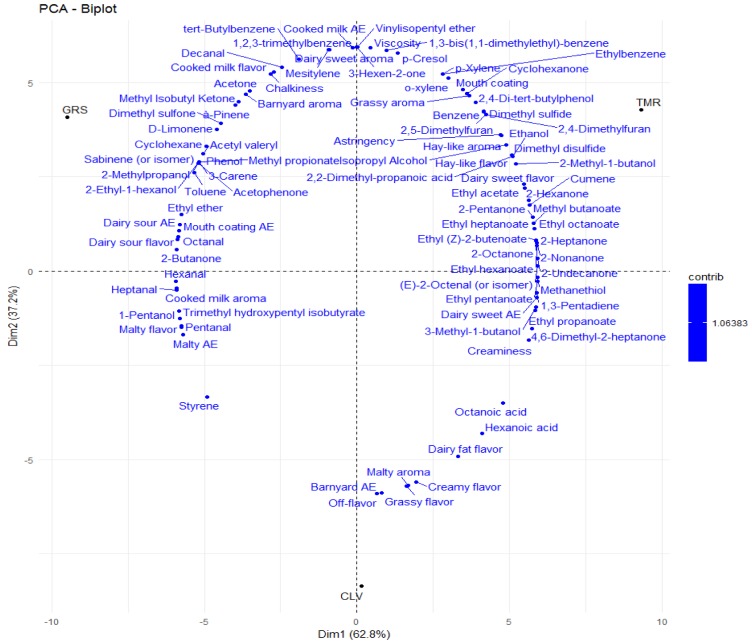
Principal component analysis (PCA) biplot of pasteurized grass (GRS), clover (CLV) and total mixed ration (TMR) milk samples showing correlations between the sensory attributes and the volatile organic compounds.

A difference in hay-like flavor was found between TMR milk and the other two milks, being significantly higher in the TMR samples. Previous studies have found that the oxidation of unsaturated fatty acids, yielding a complex mixture of volatile compounds can be involved in the formation of a hay-like flavor in food products [98,99]. Masanetz and Grosch [100] speculated that the compound 3-methyl-2,4-nonanedione could be responsible for a hay-like off-flavor in dried parsley. Interestingly, 3-methyl-2,4-nonanedione has been found to be the main contributor to the light-induced off-flavor of butter and butter oil [101]. While this specific compound was not identified in the present study, the compound 3,5-(*E*,*E*)-octadien-2-one has been described as having a grassy aroma and present at higher levels in TMR samples, this may be contributing to the hay-like flavor. Other compounds have also previously been linked to a hay-like sensory note, including, hexanal [99], 1-hexanol and *trans*-2-hexen-1-ol [102]. However, 1-hexanol was present in rTMR samples, but not pTMR samples. The thermal oxidation of vitamin A palmitate has also previously been attributed to hay-like off-flavor in non-fat milk powder [103]. Further, 2,3-pentanedione (buttery, sweet, nutty) may have been formed from 3-methyl-2,4-nonanedione through photoxidation [104] and was only detected in TMR milk samples at day nine. It is also possible that the hay-like off-flavor is being caused by a complex mixture of compounds rather than a single compound. The correlations between the sensory attributes and the VOCs are presented in Figure 6. Further variations in the volatile and sensorial profiles might be observed or accentuated in whole milk powders produced from the GRS, CLV and TMR feeding systems as the milk undergoes processing.

## 3. Materials and Methods 

### 3.1. Feed Samples 

The perennial ryegrass and perennial ryegrass/white clover samples were acquired using grass clippers cutting just above the root and were collected at 2 m intervals on a diagonal transect across each representative paddock and pooled together for each sample. Representative total mixed rations samples (mixture of grass silage, maize silage and concentrates) were taken from the cows’ feeders. Grass samples were denoted as ‘GRS feed’, grass/clover samples denoted as ‘CLV feed’ and TMR samples as ‘TMR feed’. Samples were taken at time points corresponding to the milk collections and the results were averaged. Grass-only cows (GRS) received 2 kg concentrate and 15 kg DM Grass/cow, Grass-clover (CLV) cows received 2 kg concentrate and 15 kg DM Grass-Clover/cow and TMR cows received 9 kg DM maize silage +4.5 kg DM grass silage +8.5 kg DM concentrate throughout the study. Cows within the TMR system were fed daily into electronically controlled Griffith Elder Mealmaster individual feed bins (Griffith Elder and Company Ltd., Suffolk, England) and feed was available ad-libitum. The CLV sward contained ~20% white clover as outlined by O’Callaghan, et al. [31]. Cows on pasture received a mineral supplement in the form of a liquid mineral preparation injected into the water supply (Terra Liquid Minerals, Moone Lodge, Moone, Athy, Co. Kildare, Ireland), giving a mean intake (mg/cow per d) of Na, Mg, Zn, Cu, Se, and Co of 5.0, 1.2, 219, 106, 3.8, and 3.0, respectively. The concentrate portion of the TMR feed was supplemented with a commercial mineral balancer, Dairy Hi-Phos (McDonnell Bros. Agricultural Suppliers Ltd., Fermoy, Co. Cork, Ireland) to give added Ca, Na, P, Zn, Cu, Mn, I, Co, and Se of 3340, 2000, 1200, 140, 100, 70, 10, 2, and 0.8 mg/kg, respectively [105].

### 3.2. Milk Samples and Processing

Raw milk was collected in duplicate from fifty-four spring-calving Friesian cows allocated to three experimental feeding groups (*n* = 18) based at the Teagasc Moorepark dairy farm (Fermoy, Co. Cork, Ireland) as outlined by O’Callaghan, et al. [31] at two stages of lactation (mid and late). Briefly, the milk from the cows in each of the three feeding systems; perennial ryegrass only, perennial ryegrass/white clover and TMR were separated into designated 5000-L refrigerated tanks. The evening milk was stored at 4 °C overnight, to which the morning milk was then added and agitated before collection. Late lactation pasteurized milk was used to train the sensory panel on the descriptors used for the final scoring and for the focus groups. Mid-lactation milk from each diet was used for the final scoring. Each milk sample was homogenized [GEA Niro Soavi S.p.A. Type: NS2006H (non-aseptic)] using 2-stage homogenization at 5000 to 150,000 kPa. The milk was pasteurized using a Microthermics (UHT/HTST Electric Model 25 HV Hybrid, Liquid Technologies, Wexford, Ireland) unit heated to 72 °C and held for 15 s, then cooled to 4 °C. Each milk sample was transferred at 4 °C to the sterile product outlet and aseptically packed into sterile 1-L glass bottles [1]. Pasteurization was performed within 3 h of collection, microbial analysis was performed immediately after pasteurization, and sensory analysis within one week. Samples were frozen and stored at −18 °C prior to any analysis that was not performed immediately. The volatile profile of the raw and pasteurized milk samples were analyzed at day 3, 9 and 14 of refrigerated storage in addition to FFA analysis storage period at 4 °C in order to ascertain the level of lipid oxidation occurring within the milk and to track volatile compounds forming or changing during storage. For the purpose of this study, grass milk samples are denoted as ‘GRS’, grass/clover samples as ‘CLV’ and total mixed ration as ‘TMR’. Where necessary, the prefix r is used to denote raw milk and p for pasteurized milk.

### 3.3. Microbial Analyses

Microbial analysis was performed as described by [1] with the following modifications. Each of the raw and pasteurized milk samples was plated out on three agar types; plate count skim milk agar (MPCA) to obtain the total bacteria plate count, violet red bile blood agar (VRBA) to test for the presence of coliforms and kannamycin aescilin azide agar base (KAA) to test for the presence of enterococci species. The VRBA plates were incubated at 30 °C for 24 h and the KAA plates at 37 °C for 24 h. Following incubation, all colonies that had developed were counted and the number of microorganisms per mL of milk sample was calculated.

### 3.4. Raw and Pasteurized Milk Compositions

Each milk sample was analyzed for fat, protein, lactose, true protein and casein using a Bentley DairySpec FT (Technopath Distribution, Co. Tipperary, Ireland). Samples were heated to ~40 °C in 50 mL plastic tubes (Sarstedt Ltd., Wexford, Ireland) before analysis. Results were expressed as the average of 2 replicates.

### 3.5. Free Fatty Acid Analyses

Free FA analysis was carried out on the p milk samples 3, 9 and 14 days post pasteurization. The samples were stored at 4 °C throughout analysis. Lipid extraction, methyl ester derivatization of triglycerides, solid-phase extraction (SPE), and GC instrument conditions were performed as per Mannion, et al. [106]. Further, 10 mL of each milk sample was analyzed in duplicate and the extracts were pooled for SPE.

### 3.6. Phytochemical Extraction and Analyses

Milk and feed samples from the three experimental diets (GRS, CLV and TMR) taken at two time points were pooled together for each diet, milk samples were frozen at −18 °C and feed samples were freeze dried using a Labconco stoppering tray dryer (VWR International Ltd., Dublin, Ireland). Freeze dried feed samples were milled at 10,000 rpm through a 0.5 mm mill using a Retsch Ultra Centrifugal mill ZM 200 (Lab Unlimited, Dublin, Ireland) and stored in sterile containers in a cool, dry place until required for analysis. 

The extraction procedure for milk was adapted from Antignac, et al. [107]; 10 mL of milk sample was mixed with 2 mL acetate buffer (pH 5.0; 2.0 mol/L) and 8 mL acetone for the removal of fat and protein and vortexed for 1 min and left for 16 h. The mixture was centrifuged at 435 rcf for 15 min using a Sorvall legend RT (Aquilant Scientific, Dublin, Ireland). The acetone phase was evaporated off at 45 ± 5 °C under reduced pressure to a 2-fold reduced volume using a Buchi Rotavapor R-210 (Mason Technology Ltd., Dublin, Ireland). The residue was incubated with 8 mg of a mixture of purified B-glucuronidase and sulfatase type H2 (Sigma-aldrich, Wicklow, Ireland) for 3–4 h allowing hydrolysis of the conjugated phase II metabolites followed by centrifugation at 435 rcf for 15 min. The clear supernatant was collected and applied onto C18 SPE cartridges (50 mg solid phase; Agilent Technologies Ltd., Cork, Ireland), previously activated with 6 mL methanol and 6 mL water. Following a washing step with 6 mL water, analytes were eluted with 6 mL methanol. The extract was evaporated to dryness at 45 °C under reduced pressure and reconstituted in 250 µL methanol and 250 µL 0.1 M acetate buffer (50:50, *v*/*v*) and the extracts were transferred to 1.5 mL amber vials capped with PTFE/WS 9 mm caps (Agilent Technologies Ltd., Cork, Ireland) ready for analysis. 

The extraction procedure for feed samples was adapted from Steinshamn, et al. [35]; 0.1 g of the milled feed sample was added to a mixture of methanol (3.5 mL) and 0.1 mol/L acetate buffer, pH 5.0 (1.5 mL), vortexed and left for 3–4 h. The mixture was centrifuged at 344 rcf for 15 min. The clear supernatant was evaporated to dryness at 40 ± 5 °C under reduced pressure. The residue was dissolved in 3 mL of 0.1 mol/L acetate buffer, pH 5.0 and incubated with 20 mg of cellulase and 8 mg B-glucuronidase (Sigma-Aldrich, Arklow, Co. Wicklow, Ireland) for 16 h at room temperature (~21 °C). Following a centrifugation at 455 rcf for 15 min, the extracts were transferred to 1.5 mL amber vials capped with PTFE/WS 9 mm caps (Agilent Technologies Ltd., Cork, Ireland).

Mass spectrometry profiling of the phytochemicals in the extracts was carried out on an Alliance 2695 high performance liquid chromatography unit coupled to a quadrupole time of flight mass spectrometry (HPLC-Q-Tof, Waters Corp., Milford, CT, USA). Separation of the analytes was achieved on an Atlantis T3 column 2.1 × 100 mm, 3 µm, (Waters Corp., Milford, CT, USA) using a binary solvent gradient of water containing 0.1% formic acid (solvent A) and acetonitrile containing 0.1% formic acid (solvent B). The stepwise gradient consisted of: 10% B (0–1 min), 40% B (1–6 min), 50% B (6–8 min), 70% B (8–14 min), 80% B (14–18 min) and finally back to initial gradient of 10% B at 20–25 min with flow rate of 300 µL/min. Mass spectral data were acquired in electrospray ionization mode using the following parameters: capillary voltage at 2.5 kV, cone voltage at 39 V, source temperature at 150 °C, and the desolvation temperature at 300 °C with the desolvation gas flow at 1200 L/h and mass scan range for *m*/*z* 100–1000. Accurate mass measurements of the analytes were determined using a lock mass reference leucine enkelphine (monoisoptic mass, 555.2693 Da) following the external calibration of the mass analysers using sodium formate solutions.

Quantification of the isoflavanoids was carried out on an Acquity ultra-high performance liquid chromatography-tandem quadrupole mass spectrometer (UPLC-TQD, Waters Corp., Milford, CT, USA) through multiple reaction monitoring (MRM) method. The MRM transitions of each of the four standards (apigenin, formononetin, genistein and naringenin) were generated using the Waters Intellistart^®^ software, daidzein was detected through MRM transitions. Separation of the analytes was achieved on an Acquity UPLC HSS T3 column (2.1 × 100 mm, 1.8 µm) using a binary solvent gradient of solvent A (water +0.1% formic acid) and solvent B (acetonitrile +0.1% formic acid). The solvent gradient totaling 5 min as follows: 2% B (0–0.5 min), 10% B (0.5–1.25 min), 15% B (1.25–3 min), 35% B (3.0–3.7 min), 98% B (3.7–4.7 min) and back to initial gradient of 2% B to 5 min at the flow rate of 500 µL/min was used. Data was acquired both on positive (for apigenin) and negative (for all other isoflavanoids) electrospray ionisation modes with the following settings: capillary voltage at 3 kV, cone voltage at 42 V, source temperature at 150 °C and the desolvation temperature at 350 °C with the desolvation gas flow at 1200 L/h.

### 3.7. Volatile Analyses

HS-SPME GCMS is a widely used analytical method for volatile profiling of dairy products. Volatile profiling was undertaken using a Bruker Scion 456-GC-TQ (Elementec Ltd., Maynooth, Co. Kildare, Ireland). All the incubation, extraction and injection processes were implemented using a Bruker CombiPal autosampler (Elementec Ltd., Kildare, Ireland). A mid-polar DB 624 UI column (60 m × 0.32 mm × 1.80 μm) (Agilent Technologies Ltd., Cork, Ireland) was used. A 2 cm, 50/30 μm, DVB/Carboxen/PDMS Stableflex SPME fiber (Agilent Technologies Ltd., Cork, Ireland) was selected for this study as a result of literature reviews and shown to be suitable for the extraction of volatile compounds from dairy products [108,109]. Raw and pasteurized milk samples were stored at 4 °C and analyzed in triplicate on days 3, 9 and 14. Milk (2 g) was aliquoted into amber La-Pha-Pack headspace vials (20 mL) with magnetic caps and Silicone/Polytetrafluoroethylene 1.3 mm 45° Shore A septa (Apex Scientific Ltd., Kildare, Ireland). Each sample was incubated at 40 °C with pulsed agitation for 10 min. The SPME fiber was then exposed to the headspace of the milk for 20 min while the sample was agitated. Following extraction, the SPME fiber was retracted and injected into the split/splitless 1177 GC inlet for 3 min at 250 °C in split mode at a ratio of 10:1. The column oven was held at 35 °C for 2 min, then ramped to 230 °C at a rate of 6.5 °C/min and held for 2 min and finally ramped to 260 °C at a rate of 15 °C/min and held for 5 min, yielding a total run time of 41 min. Helium was used as the carrier gas with a constant flow rate of 1.0 mL/min. Compounds were identified using an in-house library based on mass spectra obtained from NIST MS searching (v.2.3, Gaithersburg, MD, USA) and authentic standards where available. Results were processed with AMDIS software (v.2.73, Gaithersburg, MD, USA). The identification of compounds was based on target and qualifier ions and linear retention indices (LRI) [110]. An auto-tune of the GCMS system was performed regularly in order to ensure optimal GCMS performance. Air and water reports were performed prior to each run.

### 3.8. Sensory Analyses

Full descriptive sensory analysis was carried out on the three pasteurized milk samples (pGRS, pCLV and pTMR) in Teagasc Ashtown Food Research Centre (Dublin, Ireland). A 12 member external, trained descriptive sensory panel was used to assess the milk samples, using the average of seven panelist judgment’s per sample. The panel had been recruited based on their ability to perceive certain attributes and their continued availability. Panelist’s had previously received 60 h of training and had between two and three years of experience of working as descriptive panelists on a weekly basis. Training of the panel on the three milk samples consisted of two attribute generation sessions (of three hours duration each). A further four sessions of panel training took place using a variety of product standards to create aroma/texture/flavor/after effect scales for each sensory descriptor that was subsequently applied to the pGRS, pCLV, and pTMR milk samples. Panel performance assessments were carried out prior to final scoring of the three milk types. The milks were stored at 2–4 °C until approximately an hour before each training and scoring session and were allowed to reach 11–12 °C before serving. The milks were gently stirred and poured into 20 mL clear plastic cups which were labeled with random three digit codes. Panelist’s were given water and plain crackers or green apples to cleanse the palate between samples. The project was set up as a complete block design using Compusense 5.6 (sensory data capture package). All samples were scored in triplicate for each descriptor. Descriptors are outlined in Table S6 and the results are expressed as averages. Analysis of color was also carried out on each sample.

### 3.9. Statistical Analyses

Statistical analysis relating to the sensory, phytochemical and volatile data were examined using Statistical Package for the Social Sciences (SPSS) software, version 24 (IBM Corp., Armonk, NY, USA). A between- and within-subjects ANOVA with post hoc Tukey’s test were used to compare volatile compounds and sensory attribute scores of milks from herds on different feeding systems (GRS, CLV and TMR). Feeding system was the factor (independent variable) and the scores for each sensory attribute were the dependent variables. For the volatile data, feeding system was again the factor (independent variable) and the peak area responses for each volatile compound were the dependent variables. Partial least squares regression plots for the sensory results were constructed by Unscrambler Software, version 10.3 (CAMO ASA, Trondheim, Norway). The X and Y matrix was designed so that X was the sample name(s) and Y was the experimental data. Proximity of the data to the diet type (GRS, CLV, and TMR) indicated correlation between the sample and the data. The level of significance for correlation was set at *p* < 0.05 for all statistical tests unless otherwise stated. PCA biplots of the phytochemical data and the volatile vs. sensory data were constructed using the ‘factoextra’ and ‘FactoMinoR’ packages within R (v 3.4.1, R Foundation for Statistical Computing, Vienna, Austria) [111].

## 4. Conclusions

This study evaluated the effect of three widely implemented bovine feeding systems on various milk quality indicators. Significant differences were observed in volatile profile, isoflavone content and sensory perception of milk based on the feeding system. Isoflavone content was evaluated with focus on the possible breakdown products and subsequent potential effect on sensory perception. Formononetin was found to be significantly correlated to white clover feed samples and levels of apigenin, while daidzein and genistein were found to be significantly different between the r and p milk samples. Daidzein, genistein and apigenin were highly correlated to rCLV milk, likely present as metabolism products from other isoflavone compounds. Formononetin was more closely correlated with rGRS milk, despite levels of this isoflavone being higher in CLV feed. It is possible that the formonoetin in CLV feed was present in a more readily metabolised form when compared to the formonoetin content in GRS feed. Further, p-cresol is likely derived from the metabolism of formononetin and has been reported to be responsible for a barnyard aroma associated with milk derived from pasture. Both r and p GRS milk had the highest levels of p-cresol at days nine and 14 of storage, and pGRS milk was found to be more correlated with barnyard aroma than the pCLV and pTMR milk samples. Volatile profiling proved to be a useful tool for the identification of important odor-active compounds in addition to biomarkers demonstrating the authenticity of pasture-derived products. Dimethyl sulfone was identified in GRS and CLV feed and milk samples but not in TMR feed or the corresponding TMR milk samples. Most benzene compounds increased in GRS and CLV milks after pasteurization but not in TMR samples. Toluene was significantly higher in both r and p GRS and CLV milk samples throughout storage. Overall, GRS and CLV feed samples contained higher levels of alcohol compounds than TMR feed, however, this trend was not evident in the r or p milk samples suggesting the breakdown or conversion of alcohol compounds through metabolism and or pasteurization. Acid compounds were higher in TMR feed than GRS and CLV feeds. TMR feed and r and p TMR milk contained higher levels of ethyl and methyl esters, likely due to the presence of more available carbohydrate in the TMR diet combined with the presence of alcohol compounds. Full descriptive sensory analysis provided a reliable insight into the differences of the milks based on feeding system, with TMR milk having a greater white color and the GRS and CLV milk scoring higher for creamy color. Creaminess and hay-like flavor were also found to be significantly different between the p milk samples. Only the GRS and CLV milks were significantly different for creaminess, while TMR milk scored significantly highest for hay-like flavor. Results demonstrate the ability of volatile profiling and sensory techniques to distinguish milk produced from pasture versus indoor TMR feeding systems. Further research is required to ascertain the complex breakdown pathways of isoflavone compounds derived from feed and their effect on the sensory perception of bovine milk.

## Figures and Tables

**Table 1:** Concentrations of the volatile compounds identified by headspace solid-phase microextraction gas-chromatography mass spectrometry (HS-SPME GC-MS) analysis of the feed samples (grass (GRS), grass/clover (CLV) and TMR); values indicate average area values of triplicate analysis for each compound. CAS no. = Chemical Abstracts Service number. One-way ANOVA statistical analysis *** *p* < 0.001. ^1^ LRI: Linear retention index. ^2^ Ref LRI: Linear retention index reference for compounds identified by standards and/or NIST library where available.

Compound	LRI ^1^	Ref LRI ^2^	CAS No.	Grass	Grass/Clover	TMR	*p*-Value
**Aldehyde**							
2-Methyl butanal	700	700	96-17-3	6.25 × 10^8^	2.03 × 10^8^	6.29 × 10^8^	NS 0.067
3-Methyl butanal	690	692	590-86-3	5.69 × 10^8^	7.97 × 10^8^	2.28 × 10^9^	*** <0.001
Acetaldehyde	449	452	75-07-0	1.19 × 10^8^	2.73 × 10^8^	4.38 × 10^7^	*** 0.002
Butanal	627	622	123-72-8	8.78 × 10^7^	1.81 × 10^8^	0.00 × 00	*** <0.001
Decanal	1251	1256	112-31-2	4.39 × 10^9^	4.90 × 10^9^	2.08 × 10^9^	NS 0.315
Furfural	870	899	98-01-1	1.09 × 10^8^	4.31 × 10^7^	2.20 × 10^7^	*** 0.017
Heptanal	941	943	111-71-7	1.83 × 10^9^	2.75 × 10^9^	4.26 × 10^7^	*** 0.004
Hexanal	837	839	66-25-1	1.69 × 10^10^	3.59 × 10^10^	1.15 × 10^9^	*** 0.001
Nonanal	1147	1150	124-19-6	2.60 × 10^9^	5.02 × 10^9^	2.43 × 10^8^	*** 0.001
Octanal	1044	1047	124-13-0	1.47 × 10^9^	2.44 × 10^9^	0.00 × 00	*** 0.001
Pentanal	735	733	110-62-3	1.49 × 10^9^	2.07 × 10^9^	0.00 × 00	*** 0.002
Propanal	526	523	123-38-6	1.83 × 10^8^	4.92 × 10^8^	0.00 × 00	*** <0.001
Methacrolein	570	574	78-85-3	0.00 × 00	6.23 × 10^6^	0.00 × 00	*** 0.006
**Ketone**							
1-Hydroxy-2-propanone	734	734	116-09-6	0.00 × 00	2.80 × 10^8^	4.22 × 10^7^	*** 0.001
2-Butanone	638	639	78-93-3	0.00 × 00	0.00 × 00	1.08 × 10^8^	NS 0.080
2-Heptanone	932	936	110-43-0	6.22 × 10^8^	1.16 × 10^9^	2.60 × 10^8^	*** 0.011
2-Hexanone	831	834	591-78-6	1.10 × 10^8^	1.94 × 10^8^	0.00 × 00	*** 0.017
2-Nonanone	1137	1140	821-55-6	1.18 × 10^9^	1.61 × 10^9^	0.00 × 00	*** 0.010
2-Pentanone	728	730	107-87-9	5.36 × 10^8^	6.40 × 10^8^	4.79 × 10^7^	*** 0.027
4-Hydroxy-4-methyl-2-pentanone	913	913	123-42-2	4.00 × 10^9^	3.50 × 10^9^	1.43 × 10^8^	*** 0.027
6-Methyl-5-hepten-2-one	1031	1034	110-93-0	7.89 × 10^8^	2.84 × 10^9^	1.07 × 10^9^	*** <0.001
Acetoin	778	778	513-86-0	1.33 × 10^9^	1.62 × 10^9^	2.74 × 10^8^	NS 0.053
Acetone	532	533	67-64-1	3.31 × 10^8^	2.85 × 10^9^	5.43 × 10^7^	*** <0.001
Acetophenone	1141	1030	98-86-2	1.08 × 10^8^	1.77 × 10^8^	9.55 × 10^7^	NS 0.188
Acetyl valeryl (2,3-heptanedione)	875	-	96-04-8	0.00 × 00	4.32 × 10^8^	0.00 × 00	*** <0.001
Cyclohexanone	958	957	108-94-1	1.28 × 10^9^	1.33 × 10^9^	0.00 × 00	*** 0.017
Methyl Isobutyl Ketone	781	784	108-10-1	1.80 × 10^8^	0.00 × 00	0.00 × 00	*** <0.001
**Ester**							
2-Methylbutyl acetate	906	906	624-41-9	0.00 × 00	0.00 × 00	1.44 × 10^8^	NS 0.076
2-Methylbutyl butanoate	1080	-	51115-64-1	0.00 × 00	8.43 × 10^6^	9.70 × 10^8^	*** 0.005
Amyl isobutyrate (or isomer)	1121	-	2445-72-9	0.00 × 00	2.73 × 10^9^	2.60 × 10^10^	*** <0.001
Amyl propionate	992	-	105-68-0	2.59 × 10^7^	3.19 × 10^7^	6.81 × 10^8^	*** <0.001
Butyl acetate	842	842	123-86-4	0.00 × 00	0.00 × 00	9.12 × 10^8^	*** <0.001
B-Phenylethyl acetate	1339	-	103-45-7	0.00 × 00	3.75 × 10^7^	2.21 × 10^8^	*** 0.009
Dimethyl succinate	1081	1082	106-65-0	2.91 × 10^7^	2.87 × 10^7^	0.00 × 00	*** 0.019
Ethyl heptanoate	1121	-	106-30-9	6.69 × 10^7^	1.13 × 10^9^	6.42 × 10^9^	*** <0.001
Ethyl acetate	641	642	141-78-6	4.27 × 10^8^	1.06 × 10^9^	1.10 × 10^9^	*** 0.002
Ethyl benzoate	1229	-	93-89-0	7.81 × 10^7^	7.40 × 10^7^	1.37 × 10^7^	NS 0.083
Ethyl butanoate	823	826	105-54-4	0.00 × 00	2.68 × 10^9^	3.02 × 10^10^	*** <0.001
Ethyl decanoate	1420	1422	110-38-3	0.00 × 00	2.22 × 10^8^	1.45 × 10^8^	*** <0.001
Ethyl dodecanoate	1622	1621	106-33-2	0.00 × 00	1.93 × 10^8^	3.05 × 10^8^	*** <0.001
Ethyl hexanoate	1021	1024	123-66-0	2.33 × 10^9^	8.03 × 10^9^	5.44 × 10^10^	*** <0.001
Ethyl lactate	861	862	97-64-3	0.00 × 00	1.62 × 10^8^	2.60 × 10^9^	*** <0.001
Ethyl nonanoate	1319	-	123-29-5	0.00 × 00	6.26 × 10^8^	4.45 × 10^8^	NS 0.104
Ethyl octanoate	1220	-	106-32-1	4.51 × 10^8^	1.80 × 10^9^	4.76 × 10^9^	*** <0.001
Ethyl pentanoate	923	924	539-82-2	2.25 × 10^8^	1.22 × 10^9^	1.34 × 10^10^	*** <0.001
Ethyl propanoate	735	737	105-37-3	2.65 × 10^8^	7.16 ×10^8^	1.26 × 10^9^	*** <0.001
Hexyl acetate	1038	-	142-92-7	1.83 × 10^8^	3.43 × 10^8^	2.09 × 10^9^	*** <0.001
Isoamyl acetate	902	902	123-92-2	2.48 × 10^8^	3.21 × 10^8^	5.19 × 10^8^	*** 0.029
Isoamyl isobutanoate	1038	-	2050-01-3	3.07 × 10^7^	8.29 × 10^6^	2.96 × 10^9^	*** <0.001
Isobutyl butyrate	978	-	539-90-2	0.00 × 00	0.00 × 00	7.09 × 10^8^	*** <0.001
Isopentyl hexanoate	1276	-	2198-61-0	0.00 × 00	2.15 × 10^7^	6.22 × 10^8^	*** <0.001
Methyl butanoate	748	-	623-42-7	9.77 × 10^7^	9.73 × 10^8^	3.04 × 10^9^	*** <0.001
Methyl decanoate	1351	-	110-42-9	0.00 × 00	2.91 × 10^8^	0.00 × 00	*** <0.001
Methyl dodecanoate	1550	-	111-82-0	0.00 × 00	1.19 × 10^8^	6.07 × 10^7^	*** <0.001
Methyl hexanoate	949	-	106-70-7	5.52 × 10^8^	7.42 × 10^9^	1.13 × 10^10^	*** <0.001
Methyl propionate	657	-	554-12-1	1.95 × 10^7^	1.63 × 10^8^	1.08 × 10^8^	*** <0.001
n-Propyl acetate	739	-	109-60-4	1.60 × 10^9^	1.97 × 10^9^	3.43 × 10^9^	*** 0.008
Pentyl acetate	901	-	628-63-7	6.38 × 10^8^	1.72 × 10^8^	7.74 × 10^8^	*** 0.012
Propyl 2-methylbutanoate	969	-	37064-20-3	3.67 × 10^7^	5.63 × 10^7^	0.00 × 00	NS 0.112
Propyl butyrate	921	-	644-49-5	2.57 × 10^9^	3.56 × 10^9^	3.28 × 10^10^	*** <0.001
Propyl hexanoate	1118	-	626-77-7	1.53 × 10^9^	3.07 × 10^9^	3.57 × 10^10^	*** <0.001
**Alcohol**							*** <0.001
1-Hexanol	903	916	111-27-3	7.07 × 10^8^	1.95 × 10^9^	8.42 × 10^8^	NS 0.502
1-Octanol	1112	1118	111-87-5	1.56 × 10^9^	2.39 × 10^9^	0.00 × 00	*** 0.008
1-Pentanol	816	815	71-41-0	1.02 × 10^9^	1.30 × 10^9^	0.00 × 00	*** 0.018
1-Propanol	612	612	71-23-8	2.10 × 10^9^	3.68 × 10^9^	1.06 × 10^9^	*** 0.007
1-Methoxy-2-propanol	713	713	107-98-2	4.91 × 10^8^	4.80 × 10^8^	1.49 × 10^7^	*** 0.043
2-Methyl-1-butanol	783	789	137-32-6	2.75 × 10^8^	3.89 × 10^8^	2.77 × 10^8^	NS 0.418
2-Methyl-1-propanol	678	678	78-83-1	4.48 × 10^7^	8.06 × 10^7^	7.45 × 10^6^	*** 0.005
2-Methyl propanol	609	-	78-84-2	6.69 × 10^7^	7.84 × 10^7^	0.00 × 00	NS 0.112
2-Butanol	648	648	78-92-2	4.15 × 10^8^	6.63 × 10^8^	1.28 × 10^8^	*** 0.005
3-Methyl-1-butanol	783	784	123-51-3	2.41 × 10^8^	1.54 × 10^8^	2.45 × 10^7^	*** 0.043
Ethanol	506	506	64-17-5	1.23 × 10^9^	4.60 × 10^9^	1.33 × 10^9^	*** <0.001
Isopropyl Alcohol	543	-	67-63-0	6.64 × 10^7^	1.23 × 10^8^	1.80 × 10^7^	*** 0.011
Phenylethyl Alcohol	1199	-	60-12-8	0.00 × 00	9.02 × 10^7^	8.73 × 10^8^	*** <0.001
**Acid**							
2,2-Dimethyl-propanoic acid	837	869	75-98-9	0.00 × 00	2.13 × 10^8^	0.00 × 00	*** 0.004
3-Methyl-butanoic acid	918	-	503-74-2	0.00 × 00	0.00 × 00	1.04 × 10^10^	*** <0.001
Acetic acid	690	690	64-19-7	8.94 × 10^9^	1.02 × 10^10^	9.85 × 10^10^	*** <0.001
Butanoic acid	864	864	107-92-6	8.80 × 10^9^	9.17 × 10^9^	1.04 × 10^11^	*** <0.001
Pentanoic acid	995	-	109-52-4	0.00 × 00	0.00 × 00	2.51 × 10^10^	*** <0.001
Propanoic acid	778	802	79-09-4	1.51 × 10^9^	1.09 × 10^9^	2.02 × 10^10^	*** <0.001
Hexanoic acid	1052	1052	142-62-1	3.23 × 10^9^	2.90 × 10^9^	2.49 × 10^10^	*** <0.001
**Fatty acid esters**							*** <0.001
Propanoic acid, butyl ester	931	-	590-01-2	1.79 × 10^8^	0.00 × 00	9.44 × 10^8^	*** <0.001
Butanoic acid, butyl ester	978	-	109-21-7	0.00 × 00	0.00 × 00	6.04 × 10^9^	*** <0.001
**Terpene**							
3-Carene	1035	1027	13466-78-9	5.45 × 10^7^	2.41 × 10^7^	0.00 × 00	NS 0.084
α-Pinene	954	951	80-56-8	1.34 × 10^7^	0.00 × 00	8.17 × 10^6^	NS 0.270
Cumene	991	-	98-82-8	6.13 × 10^7^	1.11 × 10^8^	5.04 × 10^7^	NS 0.166
Mesitylene	1029	-	108-67-8	8.64 × 10^8^	2.03 × 10^9^	1.11 × 10^7^	*** 0.044
**Furan**							
2-Ethyl furan	717	720	3208-16-0	4.50 × 10^8^	1.25 × 10^9^	1.89 × 10^7^	*** <0.001
2-Methyl furan	615	615	534-22-5	1.64 × 10^7^	4.55 × 10^7^	0.00 × 00	*** 0.005
2-Pentyl furan	1010	1012	3777-69-3	1.12 × 10^9^	2.52 × 10^9^	5.17 × 10^8^	*** 0.004
2-n-Butyl furan	917	-	4466-24-4	0.00 × 00	6.00 × 10^7^	0.00 × 00	NS 0.079
2,4-Dimethyl furan	733	-	3710-43-8	3.27 × 10^7^	3.11 × 10^7^	0.00 × 00	*** 0.019
**Hydrocarbon**							
1,3-bis(1,1-dimethylethyl)-benzene	1284	-	1014-60-4	1.31 × 10^11^	1.73 × 10^11^	5.81 × 10^10^	NS 0.060
2,4-Dimethyl-benzaldehyde	1305	-	15764-16-6	7.46 × 10^8^	9.18 × 10^8^	2.93 × 10^8^	*** 0.019
Benzaldehyde	1027	1032	100-52-7	3.41 × 10^9^	3.44 × 10^9^	2.05 × 10^9^	NS 0.466
Benzothiazole	1320	-	95-16-9	4.06 × 10^8^	6.04 × 10^8^	5.28 × 10^8^	NS 0.156
Ethylbenzene	897	890	100-41-4	0.00 × 00	0.00 × 00	6.02 × 10^7^	*** <0.001
Mesitylene	1028	-	108-67-8	8.64 × 10^8^	2.03 × 10^9^	1.11 × 10^7^	*** 0.044
o-Cymene	1056	-	527-84-4	1.72 × 10^7^	4.73 × 10^6^	0.00 × 00	NS 0.055
o-xylene	925	916	95-47-6	1.09 × 10^8^	9.63 × 10^8^	0.00 × 00	NS 0.108
p-Xylene	895	895	106-42-3	7.52 × 10^8^	1.32 × 10^9^	5.47 × 10^8^	NS 0.146
p-Cresol	1193	-	106-44-5	4.52 × 10^8^	2.89 × 10^8^	4.40 × 10^8^	NS 0.113
Styrene	927	929	100-42-5	1.21 × 10^8^	2.37 × 10^8^	1.07 × 10^8^	NS 0.078
tert-Butylbenzene	1024	-	98-06-6	4.74 × 10^8^	6.60 × 10^8^	1.53 × 10^8^	*** 0.037
Toluene	792	794	108-88-3	8.30 × 10^7^	1.31 × 10^8^	3.62 × 10^7^	*** 0.040
**Phenolic**							
Phenol	1096	1112	108-95-2	0.00 × 00	0.00 × 00	5.87 × 10^8^	*** <0.001
2-Methoxy-4-vinylphenol	1150	-	7786-61-0	0.00 × 00	0.00 × 00	3.98 × 10^7^	*** <0.001
**Sulfur**							
Dimethyl sulfide	537	538	75-18-3	1.78 × 10^8^	2.90 × 10^8^	6.17 × 10^7^	*** 0.004
Dimethyl sulfone	1054	1055	67-71-0	2.21 × 10^8^	6.32 × 10^7^	0.00 × 00	*** 0.014
Methanethiol	459	462	74-93-1	9.29 × 10^6^	1.02 × 10^7^	8.84 × 10^6^	NS 0.855
**Ether**							
Vinylisopentyl ether	765	-	39782-38-2	1.10 × 10^8^	2.89 × 10^8^	3.71 × 10^7^	*** 0.033
**Lactone**							
y-Hexalactone	1166	-	695-06-7	8.51 × 10^8^	1.07 × 10^9^	4.06 × 10^8^	*** 0.040
y-Nonalactone	1489	-	104-61-0	4.69 × 10^8^	6.44 × 10^8^	3.75 × 10^8^	NS 0.279
**Pyrazine**							
2,3,5-Trimethyl-6-ethylpyrazine	1190	-	17398-16-2	0.00 × 00	0.00 × 00	1.48 × 10^8^	*** <0.001
2,3-Dimethyl-pyrazine	961	-	5910-89-4	1.27 × 10^8^	3.77 × 10^7^	1.33 × 10^9^	*** <0.001
3-Ethyl-2,5-dimethyl-pyrazine	1055	-	5910-89-4	0.00 × 00	0.00 × 00	4.71 × 10^7^	*** <0.001
Pyrazine	771	-	290-37-9	5.46 × 10^7^	5.55 × 10^7^	4.79 × 10^7^	NS 0.847
Trimethyl-pyrazine	1041	1041	14667-55-1	8.50 × 10^7^	3.89 × 10^7^	9.03 × 10^8^	*** <0.001

**Table 2:** Relationship between cow feeding system (grass, grass/clover and total mixed ration (TMR)) and the raw (r) milk volatile compounds identified by headspace solid-phase microextraction gas-chromatography mass spectrometry (HS-SPME GC-MS) at day 3, 9 and 14 of refrigerated storage; values are expressed as peak area values for each compound. d = day, * *p* = 0.05, ND = not detected, NS = not significant, GRS = Grass, CLV = Grass/clover. ^1^ LRI = Linear retention index.

Compound	CAS No.	LRI ^1^	Grass d 3	Grass/Clover d 3	TMR d 3	Grass d 9	Grass/Clover d 9	TMR Day 9	Grass d 14	Grass/Clover d 14	TMR Day 14	*p*-Value	*p*-Value (Grass)	*p*-Value (Grass/Clover)	*p*-Value (TMR)
**Aldehyde**															
(E)-2-Octenal (or isomer)	2548-87-0	1094	0.00 × 00	0.00 × 00	1.74 × 10^7^	7.29 × 10^7^	2.51 × 10^8^	2.22 × 10^7^	2.85 × 10^8^	1.11 × 10^9^	2.18 × 10^8^	* <0.001	NS 0.051	* 0.003	* 0.05
(Z)-2-Heptenal (or isomer)	57266-86-1	1012	0.00 × 00	0.00 × 00	0.00 × 00	0.00 × 00	0.00 × 00	2.03 × 10^7^	0.00 × 00	0.00 × 00	0.00 × 00	* <0.001	ND	ND	* 0.006
Acetaldehyde	75-07-0	449	0.00 × 00	0.00 × 00	0.00 × 00	0.00 × 00	0.00 × 00	0.00 × 00	2.85 × 10^7^	0.00 × 00	2.40 × 10^6^	* 0.029	NS 0.302	ND	NS 0.129
3-Methyl-butanal	590-86-3	690	0.00 × 00	0.00 × 00	0.00 × 00	0.00 × 00	0.00 × 00	0.00 × 00	9.93 × 10^7^	7.12 × 10^8^	1.75 × 10^7^	* <0.001	* <0.001	* <0.001	* 0.002
Decanal	112-31-2	1250	1.47 × 10^7^	1.29 × 10^7^	3.26 × 10^6^	8.28 × 10^6^	5.23 × 10^6^	4.97 × 10^6^	1.18 × 10^7^	3.73 × 10^6^	3.52 × 10^6^	NS 0.477	NS 0.658	NS 0.515	NS 0.736
Heptanal	111-71-7	941	1.09 × 10^8^	1.08 × 10^8^	1.49 × 10^8^	1.06 × 10^7^	1.72 × 10^6^	7.27 × 10^8^	0.00 × 00	0.00 × 00	0.00 × 00	* <0.001	* 0.001	* <0.001	* <0.001
Hexanal	66-25-1	838	3.69 × 10^8^	4.02 × 10^8^	1.72 × 10^9^	0.00 × 00	0.00 × 00	3.56 × 10^9^	0.00 × 00	0.00 × 00	0.00 × 00	* <0.001	* 0.000	* <0.001	* <0.001
Nonanal	124-19-6	1147	5.31 × 10^7^	5.02 × 10^7^	6.91 × 10^7^	3.66 × 10^7^	2.81 × 10^7^	1.23 × 10^8^	3.98 × 10^7^	1.90 × 10^7^	3.44 × 10^7^	* <0.001	NS 0.259	* <0.001	* <0.001
Octanal	124-13-0	1044	2.22 × 10^7^	3.11 × 10^7^	4.02 × 10^7^	1.21 × 10^7^	0.00 × 00	8.73 × 10^7^	0.00 × 00	0.00 × 00	0.00 × 00	* <0.001	* 0.007	* 0.009	* <0.001
Pentanal	110-62-3	733	1.39 × 10^8^	1.96 × 10^8^	9.84 × 10^6^	0.00 × 00	0.00 × 00	1.82 × 10^8^	0.00 × 00	0.00 × 00	0.00 × 00	* <0.001	* <0.001	* <0.001	* <0.001
**Ketone**															
2-Butanone	78-93-3	637	3.86 × 10^7^	1.05 × 10^8^	1.49 × 10^8^	5.86 × 10^7^	1.11 × 10^8^	1.48 × 10^8^	2.64 × 10^7^	9.64 × 10^7^	7.42 × 10^7^	* <0.001	NS 0.127	* 0.007	* <0.001
2-Heptanone	110-43-0	933	3.37 × 10^7^	3.60 × 10^7^	3.20 × 10^7^	4.37 × 10^8^	8.14 × 10^8^	4.67 × 10^7^	1.52 × 10^9^	3.63 × 10^9^	9.04 × 10^9^	* <0.001	* <0.001	* <0.001	* <0.001
2-Hexanone	591-78-6	831	1.77 × 10^7^	9.14 × 10^6^	8.93 × 10^6^	2.33 × 10^7^	2.96 × 10^7^	2.35 × 10^6^	2.63 × 10^7^	6.70 × 10^7^	8.27 × 10^7^	* <0.001	NS 0.603	* <0.001	* <0.001
2-Nonanone	821-55-6	1137	0.00 × 00	0.00 × 00	0.00 × 00	2.25 × 10^8^	1.49 × 10^8^	0.00 × 00	5.16 × 10^8^	5.69 × 10^8^	2.46 × 10^9^	* <0.001	* 0.005	* 0.002	* <0.001
2-Octanone	111-13-7	1034	6.82 × 10^6^	1.01 × 10^7^	1.13 × 10^7^	1.04 × 10^7^	2.62 × 10^7^	2.21 × 10^6^	2.31 × 10^7^	5.00 × 10^7^	5.10 × 10^7^	* <0.001	* 0.027	* <0.001	* 0.001
2-Pentanone	107-87-9	727	5.70 × 10^7^	5.47 × 10^7^	5.36 × 10^7^	1.06 × 10^8^	2.17 × 10^8^	4.00 × 10^7^	1.94 × 10^8^	5.48 × 10^8^	6.91 × 10^8^	* <0.001	* <0.001	* <0.001	* <0.001
2-Undecanone	112-12-9	1353	0.00 × 00	0.00 × 00	0.00 × 00	5.97 × 10^6^	4.63 × 10^5^	0.00 × 00	3.82 × 10^7^	1.52 × 10^7^	2.78 × 10^8^	* <0.001	NS 0.262	* 0.047	* <0.001
2,3-Pentanedione	600-14-6	736	0.00 × 00	0.00 × 00	0.00 × 00	0.00 × 00	0.00 × 00	2.03 × 10^8^	0.00 × 00	0.00 × 00	0.00 × 00	* <0.001	ND	ND	* 0.007
3,5-(*E*,*E*)-Octadien-2-one (or isomer)	30086-02-3	1130	0.00 × 00	0.00 × 00	0.00 × 00	0.00 × 00	0.00 × 00	1.17 × 10^7^	0.00 × 00	0.00 × 00	0.00 × 00	* <0.001	ND	ND	* 0.015
3-Hexen-2-one	763-93-9	839	6.97 × 10^6^	7.68 × 10^6^	2.76 × 10^6^	1.59 × 10^7^	1.54 × 10^7^	0.00 × 00	1.53 × 10^7^	8.90 × 10^6^	1.58 × 10^7^	NS 0.177	NS 0.557	NS 0.517	NS 0.051
4-Methyl-3-pentene-2-one (tentative)	141-79-7	839	0.00 × 00	0.00 × 00	0.00 × 00	1.15 × 10^7^	5.55 × 10^6^	0.00 × 00	9.92 × 10^6^	1.23 × 10^7^	1.58 × 10^7^	* 0.047	NS 0.500	ND 0.065	* 0.025
5-Hepten-2-one (tentative)	6714-00-7	921	0.00 × 00	0.00 × 00	0.00 × 00	0.00 × 00	0.00 × 00	0.00 × 00	0.00 × 00	2.42 × 10^7^	5.23 × 10^7^	* <0.001	ND	* <0.001	* <0.001
Acetone	67-64-1	532	9.16 × 10^8^	8.04 × 10^8^	1.14 × 10^9^	7.81 × 10^8^	7.97 × 10^8^	1.22 × 10^9^	3.00 × 10^8^	7.20 × 10^8^	6.65 × 10^8^	* <0.001	* 0.003	NS 0.538	* <0.001
Acetophenone	98-86-2	1030	6.38 × 10^6^	2.05 × 10^6^	1.64 × 10^6^	0.00 × 00	4.23 × 10^6^	3.22 × 10^6^	1.77 × 10^6^	1.06 × 10^6^	0.00 × 00	* 0.044	NS 0.114	NS 0.113	NS 0.251
Cyclohexanone	110-82-7	956	7.99 × 10^6^	0.00 × 00	0.00 × 00	1.37 × 10^7^	1.67 × 10^6^	0.00 × 00	6.52 × 10^6^	1.33 × 10^6^	1.33 × 10^6^	* 0.046	NS 0.728	NS 0.623	NS 0.422
Acetyl valeryl (2,3-heptanedione)	96-04-8	875	3.78 × 10^5^	2.52 × 10^5^	4.29 × 10^5^	3.31 × 10^6^	3.56 × 10^7^	1.77 × 10^5^	0.00 × 00	2.39 × 10^7^	1.31 × 10^6^	* <0.001	NS 0.194	* 0.001	NS 0.582
Methyl Isobutyl Ketone	108-10-1	780	2.05 × 10^8^	1.47 × 10^8^	2.00 × 10^8^	2.14 × 10^8^	1.73 × 10^8^	2.46 × 10^8^	1.74 × 10^8^	1.78 × 10^8^	1.75 × 10^8^	* <0.001	* 0.035	* 0.023	* 0.006
**Ester**															
Ethyl heptanoate	106-30-9	1120	0.00 × 00	0.00 × 00	0.00 × 00	0.00 × 00	0.00 × 00	0.00 × 00	4.08 × 10^7^	0.00 × 00	1.35 × 10^8^	* <0.001	* <0.001	ND	* <0.001
Ethyl (Z)-2-butenoate	6776-19-8	875	0.00 × 00	0.00 × 00	0.00 × 00	0.00 × 00	0.00 × 00	0.00 × 00	4.12 × 10^8^	2.51 × 10^6^	1.54 × 10^8^	* <0.001	* <0.001	NS 0.422	* <0.001
Ethyl 2-methylbutanoate	7452-79-1	872	0.00 × 00	0.00 × 00	0.00 × 00	0.00 × 00	0.00 × 00	0.00 × 00	2.38 × 10^7^	0.00 × 00	0.00 × 00	* <0.001	* <0.001	ND	ND
Ethyl 3-methylbutanoate	108-64-5	876	0.00 × 00	0.00 × 00	0.00 × 00	0.00 × 00	0.00 × 00	0.00 × 00	1.81 × 10^8^	0.00 × 00	0.00 × 00	* <0.001	* <0.001	ND	ND
Ethyl acetate	141-78-6	639	0.00 × 00	0.00 × 00	0.00 × 00	2.02 × 10^7^	8.47 × 10^6^	0.00 × 00	2.44 × 10^8^	6.08 × 10^7^	1.85 × 10^8^	* <0.001	* <0.001	* <0.001	* <0.001
Ethyl butanoate	105-54-4	823	0.00 × 00	0.00 × 00	0.00 × 00	0.00 × 00	0.00 × 00	0.00 × 00	6.10 × 10^9^	0.00 × 00	1.02 × 10^10^	* <0.001	* <0.001	ND	* <0.001
Ethyl decanoate	110-38-3	1419	0.00 × 00	0.00 × 00	0.00 × 00	0.00 × 00	0.00 × 00	0.00 × 00	4.30 × 10^8^	9.37 × 10^6^	1.59 × 10^9^	* <0.001	* 0.019	NS 0.155	* <0.001
Ethyl hexanoate	123-66-0	1021	0.00 × 00	0.00 × 00	0.00 × 00	4.18 × 10^8^	0.00 × 00	0.00 × 00	6.90 × 10^9^	2.80 × 10^7^	8.91 × 10^9^	* <0.001	* <0.001	* 0.005	* <0.001
Ethyl octanoate	106-32-1	1220	0.00 × 00	0.00 × 00	0.00 × 00	0.00 × 00	0.00 × 00	0.00 × 00	7.85 × 10^8^	3.72 × 10^6^	3.07 × 10^9^	* <0.001	* <0.001	NS 0.105	* <0.001
Ethyl pentanoate	539-82-2	923	0.00 × 00	0.00 × 00	0.00 × 00	0.00 × 00	0.00 × 00	0.00 × 00	5.50 × 10^7^	0.00 × 00	8.08 × 10^7^	* <0.001	* 0.001	ND	* <0.001
Ethyl propanoate	105-37-3	735	0.00 × 00	0.00 × 00	0.00 × 00	0.00 × 00	0.00 × 00	0.00 × 00	4.73 × 10^6^	0.00 × 00	2.51 × 10^7^	* <0.001	NS 0.465	ND	* <0.001
Methyl butanoate	623-42-7	747	0.00 × 00	0.00 × 00	0.00 × 00	2.25 × 10^6^	4.90 × 10^6^	0.00 × 00	9.84 × 10^5^	2.78 × 10^7^	1.47 × 10^7^	* <0.001	NS 0.590	* <0.001	* <0.001
Methyl decanoate	110-42-9	1350	0.00 × 00	0.00 × 00	0.00 × 00	0.00 × 00	0.00 × 00	0.00 × 00	0.00 × 00	0.00 × 00	2.52 × 10^6^	NS 0.084	ND	ND	NS 0.143
Methyl hexanoate	106-70-7	949	0.00 × 00	0.00 × 00	0.00 × 00	1.34 × 10^6^	0.00 × 00	0.00 × 00	3.65 × 10^6^	0.00 × 00	3.00 × 10^7^	* <0.001	NS 0.244	ND	* <0.001
Methyl methacrylate	80-62-6	736	7.47 × 10^6^	2.22 × 10^6^	3.88 × 10^6^	0.00 × 00	0.00 × 00	0.00 × 00	0.00 × 00	0.00 × 00	0.00 × 00	* 0.002	* <0.001	NS 0.422	* <0.001
**Alcohol**															
1-Butanol	71-36-3	715	0.00 × 00	0.00 × 00	0.00 × 00	0.00 × 00	4.08 × 10^6^	7.33 × 10^5^	0.00 × 00	0.00 × 00	0.00 × 00	* 0.040	ND	NS 0.080	NS 0.422
2-Methyl-1-butanol	137-32-6	765	2.27 × 10^7^	2.15 × 10^7^	1.97 × 10^7^	2.23 × 10^7^	2.02 × 10^7^	1.95 × 10^7^	0.00 × 00	0.00 × 00	1.18 × 10^8^	* <0.001	* 0.003	* 0.003	* <0.001
3-Methyl-1-butanol	123-51-3	767	5.73 × 10^7^	6.92 × 10^7^	1.11 × 10^8^	5.48 × 10^7^	3.01 × 10^7^	5.04 × 10^7^	1.05 × 10^9^	2.56 × 10^9^	3.33 × 10^8^	* <0.001	* 0.011	* <0.001	* <0.001
1-Hexanol	111-27-3	894	0.00 × 00	0.00 × 00	5.83 × 10^7^	0.00 × 00	0.00 × 00	0.00 × 00	0.00 × 00	0.00 × 00	0.00 × 00	* <0.001	ND	NS 0.080	* 0.004
2-Ethyl-1-hexanol	104-76-7	1075	3.69 × 10^7^	3.34 × 10^7^	1.96 × 10^7^	0.00 × 00	0.00 × 00	0.00 × 00	0.00 × 00	0.00 × 00	0.00 × 00	* <0.001	* <0.001	* 0.001	NS 0.108
1-Octanol	111-87-5	1116	0.00 × 00	0.00 × 00	2.06 × 10^7^	0.00 × 00	0.00 × 00	2.22 × 10^7^	0.00 × 00	0.00 × 00	0.00 × 00	* <0.001	ND	ND	* 0.012
1-Pentanol	71-41-0	794	6.10 × 10^7^	9.15 × 10^7^	1.57 × 10^7^	2.95 × 10^7^	5.39 × 10^7^	8.62 × 10^7^	0.00 × 00	2.52 × 10^6^	2.52 × 10^7^	* <0.001	NS 0.075	* <0.001	* <0.001
Ethanol	64-17-5	505	0.00 × 00	0.00 × 00	0.00 × 00	0.00 × 00	1.35 × 10^7^	0.00 × 00	2.10 × 10^8^	4.36 × 10^8^	1.14 × 10^9^	* 0.000	* 0.005	NS 0.070	* 0.003
Isopropyl Alcohol	67-63-0	541	0.00 × 00	0.00 × 00	0.00 × 00	2.54 × 10^7^	2.33 × 10^7^	0.00 × 00	4.29 × 10^7^	2.82 × 10^7^	6.11 × 10^7^	* <0.001	NS 0.194	NS 0.146	* <0.001
**Acid**															
Butanoic acid	107-92-6	863	0.00 × 00	0.00 × 00	0.00 × 00	0.00 × 00	0.00 × 00	0.00 × 00	0.00 × 00	0.00 × 00	9.61 × 10^8^	NS 0.526	ND	ND	NS 0.385
Hexanoic acid	142-62-1	1052	0.00 × 00	0.00 × 00	0.00 × 00	0.00 × 00	0.00 × 00	0.00 × 00	2.70 × 10^8^	0.00 × 00	6.72 × 10^9^	NS 0.371	NS 0.259	ND	NS 0.306
Octanoic acid	124-07-2	1245	0.00 × 00	0.00 × 00	0.00 × 00	0.00 × 00	0.00 × 00	0.00 × 00	0.00 × 00	0.00 × 00	1.67 × 10^9^	NS 0.471	ND	ND	NS 0.358
Propanoic acid, 2-methyl-, 3-hydroxy-2,2,4-trimethylpentyl ester	77-68-9	1460	6.78 × 10^7^	5.62 × 10^7^	3.46 × 10^7^	1.62 × 10^7^	2.71 × 10^7^	1.01 × 10^7^	3.01 × 10^7^	5.71 × 10^6^	7.09 × 10^5^	* 0.003	NS 0.026	* 0.015	NS 0.181
**Terpene**															
3-Carene	13466-78-9	1035	0.00 × 00	0.00 × 00	1.34 × 10^7^	0.00 × 00	9.50 × 10^5^	7.02 × 10^6^	0.00 × 00	0.00 × 00	0.00 × 00	NS 0.144	ND	NS 0.144	NS 0.285
α-Pinene	80-56-8	953	7.81 × 10^6^	6.03 × 10^6^	4.56 × 10^6^	7.50 × 10^7^	6.39 × 10^7^	4.88 × 10^7^	1.62 × 10^7^	2.73 × 10^7^	1.10 × 10^7^	* <0.001	* 0.001	* 0.012	* 0.003
Cumene	98-82-8	990	2.26 × 10^6^	2.80 × 10^6^	3.47 × 10^6^	5.58 × 10^6^	7.86 × 10^6^	3.13 × 10^6^	3.71 × 10^6^	1.06 × 10^7^	1.64 × 10^7^	* 0.035	NS 0.323	NS 0.220	* 0.037
D-Limonene	5989-27-5	1055	0.00 × 00	2.78 × 10^7^	1.61 × 10^7^	1.26 × 10^6^	3.05 × 10^5^	1.77 × 10^7^	0.00 × 00	0.00 × 00	0.00 × 00	* <0.001	NS 0.465	* <0.001	0.013
Mesitylene	108-67-8	1028	4.47 × 10^7^	3.71 × 10^7^	5.38 × 10^7^	5.14 × 10^7^	4.54 × 10^7^	2.98 × 10^7^	7.23 × 10^7^	6.79 × 10^7^	8.22 × 10^7^	* 0.017	NS 0.104	NS 0.070	* 0.042
trans-β-Ocimene (or isomer)	3779-61-1	1035	0.00 × 00	1.74 × 10^7^	1.34 × 10^7^	0.00 × 00	0.00 × 00	1.77 × 10^7^	0.00 × 00	0.00 × 00	0.00 × 00	* 0.020	ND	NS 0.088	NS 0.067
**Furan**															
2,4-Dimethylfuran	3710-43-8	732	8.90 × 10^6^	3.64 × 10^6^	7.27 × 10^6^	1.11 × 10^7^	1.36 × 10^7^	0.00 × 00	8.34 × 10^6^	1.56 × 10^7^	1.17 × 10^7^	* <0.001	NS 0.357	* <0.001	* <0.001
2,5-Dimethylfuran	625-86-5	734	8.90 × 10^6^	3.64 × 10^6^	7.27 × 10^6^	1.11 × 10^7^	1.36 × 10^7^	0.00 × 00	8.34 × 10^6^	1.56 × 10^7^	1.17 × 10^7^	* <0.001	NS 0.357	* 0.002	* <0.001
2-Ethylfuran	3208-16-0	717	0.00 × 00	0.00 × 00	0.00 × 00	0.00 × 00	0.00 × 00	3.04 × 10^6^	0.00 × 00	0.00 × 00	0.00 × 00	NS 0.090	ND	NS 0.422	NS 0.172
**Hydrocarbon**															
2,6-Bis(1,1-dimethylethyl)-4-(1-oxopropyl)phenol (tentative)	14035-34-8	1684	1.13 × 10^7^	1.88 × 10^7^	1.35 × 10^7^	2.41 × 10^7^	3.89 × 10^7^	2.39 × 10^7^	5.18 × 10^6^	1.63 × 10^7^	2.83 × 10^7^	* 0.012	NS 0.347	* 0.008	NS 0.171
2,4-Dimethyl-benzaldehyde	15764-16-6	1305	6.81 × 10^6^	2.24 × 10^6^	2.48 × 10^6^	0.00 × 00	0.00 × 00	0.00 × 00	0.00 × 00	0.00 × 00	0.00 × 00	* <0.001	* <0.001	* 0.028	* 0.028
Benzene	71-43-2	684	2.55 × 10^6^	2.57 × 10^6^	4.69 × 10^6^	3.63 × 10^6^	8.95 × 10^7^	1.98 × 10^5^	3.21 × 10^6^	4.13 × 10^7^	2.80 × 10^6^	* <0.001	NS 0.943	* 0.017	NS 0.232
1,2,3-Trimethyl-benzene	526-73-8	1028	4.47 × 10^7^	3.71 × 10^7^	5.38 × 10^7^	5.14 × 10^7^	4.54 × 10^7^	2.98 × 10^7^	7.23 × 10^7^	6.79 × 10^7^	8.22 × 10^7^	* 0.017	NS 0.104	NS 0.070	* 0.042
1,3-Bis(1,1-dimethylethyl)-benzene	1014-60-4	1284	3.49 × 10^8^	3.08 × 10^8^	2.33 × 10^8^	3.67 × 10^8^	3.10 × 10^8^	2.01 × 10^8^	4.52 × 10^8^	4.41 × 10^8^	6.52 × 10^8^	* <0.001	NS 0.185	* 0.007	* <0.001
Ethylbenzene	100-41-4	897	1.01 × 10^8^	8.28 × 10^7^	9.66 × 10^7^	1.69 × 10^8^	2.08 × 10^8^	7.05 × 10^7^	2.07 × 10^8^	3.05 × 10^8^	3.32 × 10^8^	* <0.001	* 0.004	* <0.001	* <0.001
o-Cymene	527-84-4	1055	0.00 × 00	0.00 × 00	0.00 × 00	5.76 × 10^6^	3.30 × 10^6^	0.00 × 00	0.00 × 00	0.00 × 00	0.00 × 00	* <0.001	* <0.001	NS 0.128	ND
p-Cresol	106-44-5	1182	0.00 × 00	0.00 × 00	0.00 × 00	8.49 × 10^8^	3.62 × 10^8^	2.48 × 10^8^	6.35 × 10^7^	5.22 × 10^7^	5.46 × 10^7^	* <0.001	* 0.003	* <0.001	* <0.001
p-Xylene	106-42-3	895	1.01 × 10^8^	8.28 × 10^7^	9.66 × 10^7^	1.69 × 10^8^	2.08 × 10^8^	7.05 × 10^7^	2.07 × 10^8^	3.05 × 10^8^	3.32 × 10^8^	* <0.001	* 0.004	* <0.001	* <0.001
Styrene	100-42-5	927	0.00 × 00	0.00 × 00	0.00 × 00	6.15 × 10^6^	0.00 × 00	2.92 × 10^6^	4.11 × 10^6^	0.00 × 00	8.62 × 10^6^	NS 0.223	NS 0.631	ND	NS 0.214
tert-Butylbenzene	98-06-6	1024	9.63 × 10^6^	8.24 × 10^6^	6.27 × 10^6^	8.48 × 10^6^	6.51 × 10^6^	3.21 × 10^6^	8.80 × 10^6^	1.23 × 10^7^	1.32 × 10^7^	NS 0.072	NS 0.815	NS 0.223	* 0.044
Toluene	108-88-3	792	1.94 × 10^9^	1.27 × 10^9^	4.98 × 10^7^	1.97 × 10^9^	1.41 × 10^9^	4.51 × 10^7^	1.59 × 10^9^	1.22 × 10^9^	4.97 × 10^7^	* <0.001	NS 0.075	* 0.008	NS 0.695
**Phenolic**															
Phenol	108-95-2	1093	0.00 × 00	0.00 × 00	0.00 × 00	4.65 × 10^6^	0.00 × 00	5.29 × 10^6^	9.74 × 10^6^	0.00 × 00	5.77 × 10^6^	* 0.021	NS 0.205	ND	NS 0.220
2,4-Di-tert-butylphenol	96-76-4	1595	0.00 × 00	0.00 × 00	0.00 × 00	5.79 × 10^7^	1.39 × 10^7^	1.38 × 10^7^	2.37 × 10^7^	2.00 × 10^6^	1.01 × 10^7^	* 0.043	NS 0.363	NS 0.065	NS 0.130
**Sulfur**															
Dimethyl sulfide	75-18-3	536	1.11 × 10^7^	1.05 × 10^7^	6.30 × 10^6^	2.39 × 10^7^	1.75 × 10^7^	0.00 × 00	1.66 × 10^8^	1.38 × 10^8^	5.51 × 10^7^	* <0.001	* <0.001	* <0.001	* <0.001
Dimethyl sulfone	67-71-0	1052	3.96 × 10^7^	2.94 × 10^7^	0.00 × 00	3.88 × 10^7^	2.80 × 10^7^	0.00 × 00	3.60 × 10^7^	2.41 × 10^7^	0.00 × 00	* <0.001	NS 0.961	NS 0.675	ND
Dimethyl disulfide	624-92-0	776	0.00 × 00	0.00 × 00	0.00 × 00	0.00 × 00	0.00 × 00	0.00 × 00	0.00 × 00	0.00 × 00	0.00 × 00	* 0.033	ND	NS 0.093	NS 0.422
Methanethiol	74-93-1	459	0.00 × 00	0.00 × 00	0.00 × 00	0.00 × 00	0.00 × 00	0.00 × 00	0.00 × 00	4.79 × 10^7^	0.00 × 00	* <0.001	ND	* 0.013	ND
**Ether**															
Ethyl ether	60-29-7	514	1.86 × 10^7^	1.09 × 10^7^	6.58 × 10^6^	0.00 × 00	0.00 × 00	0.00 × 00	0.00 × 00	0.00 × 00	0.00 × 00	* 0.006	NS 0.013	NS 0.086	NS 0.232
Vinylisopentyl ether	39782-38-2	767	5.73 × 10^7^	7.61 × 10^7^	7.42 × 10^7^	4.76 × 10^7^	3.01 × 10^7^	2.71 × 10^7^	0.00 × 00	0.00 × 00	1.35 × 10^8^	NS 0.377	NS 0.645	NS 0.234	NS 0.337

## Supplementary Materials

The following are available online at https://www.mdpi.com/1420-3049/25/1/26/s1, Table S1: Microbial results for the raw and pasteurized milk samples (GRS, CLV and TMR). VRB: Violet Red Bile, KAA: Kanamycin Aesculin Azide, MPCA: Milk Plate Count Agar, Table S2: Composition analysis results for pasteurized grass (GRS), clover (CLV) and TMR milk samples in early, mid and late lactation. Each result is the average of 2 replicates, Table S3: Individual Free Fatty Acid Content mg/kg or ppm (relative standard deviation of the results between replicates as a percent in brackets) for each of the p milk samples (GRS, CLV and TMR) at day 3, 9 and 14 of refrigerated storage. * *p* = 0.05, d = day, Table S4: Individual Free fatty acid content mg/kg (relative standard deviation of the results between replicates as a percent in brackets) for each of the p milk samples (GRS, CLV and TMR) at day 3, 9 and 14 of refrigerated storage and the significance between the fatty acids analyzed for each sample (GRS, CLV and TMR). * *p* = 0.05, d = day, NS = not significant, Figure S1: Bar chart showing the levels of important isoflavones in feed samples (grass, Grass/clover and TMR) and the corresponding raw (r) and pasteurized (p) milk samples. Grass [GRS], grass/clover [CLV]; Figure S2: Hierarchal clustering analysis (Heatmap) of the average values for the top 65 volatile organic compounds contributing to the differences between grass, grass/clover and TMR feed samples, as determined by headspace solid-phase microextraction GC-MS analysis. Positive and negative correlations between feeding system (grass, grass/clover and TMR) and volatile organic compounds is denoted by +1 (red) and −1 (blue), Figure S3: Bar charts showing the percentage of each chemical class (aldehydes, ketones, alcohols, acids, fatty acid esters, terpenes, furans, hydrocarbons, sulphurs, lactones, pyrazines, ether and phenol) identified in each feed type (grass, grass/clover and TMR). 90, 104 and 94 compounds were identified in grass, grass/clover and TMR feeds, respectively. Table S5: Relationship between cow diet (Grass, Clover and TMR) and the pasteurized milk (p) volatile compounds identified by HS SPME GC-MS at day 3, 9 and 14 of refrigerated storage; values are expressed as peak area values for each compound.; values are expressed as peak area values for each compound. d = day, * *p* = 0.05, ND = not detected, NS = not significant, Table S6: The 26 sensory descriptors used for the evaluation of the 3 pasteurized milk samples (GRS, CLV and TMR) by full descriptive sensory analysis.
