# Supplementary material for: Dietary Compounds Influencing the Sensorial, Volatile and Phytochemical Properties of Bovine Milk
Source: Molecules. 2019 Dec 19;25(1):26. doi: 10.3390/molecules25010026 (PMC6983252; doi:10.3390/molecules25010026)
Supplement: Supplementary file 1 [file molecules-25-00026-s001.pdf]

Supplementary Materials

Table S1: Microbial results for the raw and pasteurized milk samples (grass (GRS), clover (CLV) and total mixed ration (TMR)). VRB: Violet Red Bile, KAA: Kanamycin Aesculin Azide, MPCA: Milk Plate Count Agar.

Table S2: Composition analysis results for pasteurized grass (GRS), clover (CLV) and total mixed ration (TMR) milk samples in early, mid and late lactation. Each result is the average of 2 replicates.

Table S3: Individual Free Fatty Acid Content mg/kg or ppm (relative standard deviation of the results between replicates as a percent in brackets) for each of the pasteurized (p) milk samples (grass (GRS), grass/clover (CLV) and total mixed ration (TMR)) at day 3, 9 and 14 of refrigerated storage. * $p = 0.05$, d = day.

Table S4: Individual Free fatty acid content mg/kg (relative standard deviation of the results between replicates as a percent in brackets) for each of the pasteurized (p) milk samples (grass (GRS), grass/clover (CLV) and total mixed ration (TMR)) at day 3, 9 and 14 of refrigerated storage and the significance between the fatty acids analysed for each sample (GRS, CLV and TMR). * $p = 0.05$, d = day, NS = not significant.

Figure S1: Bar chart showing the levels of important isoflavones in feed samples (grass, grass/clover and total mixed ration (TMR)) and the corresponding raw (r) and pasteurized (p) grass (GRS), clover (CLV) and total mixed ration (TMR) milk samples.

Figure S2: Hierarchal clustering analysis (Heatmap) of the average values for the top 65 volatile organic compounds contributing to the differences between grass, grass/clover and total mixed ration (TMR) feed samples, as determined by headspace solid-phase microextraction gas-chromatography mass spectrometry (HS-SPME GC-MS) analysis. Positive and negative correlations between feeding system (grass, grass/clover and TMR) and volatile organic compounds is denoted by +1 (red) and −1 (blue).

Figure S3: Bar charts showing the percentage of each chemical class (aldehydes, ketones, alcohols, acids, fatty acid esters, terpenes, furans, hydrocarbons, sulphurs, lactones, pyrazines, ether and phenol) identified in each feed type (grass, grass/clover and total mixed ration (TMR)). 90, 104 and 94 compounds were identified in grass, grass/clover and TMR feeds, respectively.

Table S5: Relationship between cow diet (grass, grass/clover and total mixed ration (TMR)) and the pasteurized (p) milk volatile compounds identified by HS SPME GC-MS at day 3, 9 and 14 of refrigerated storage; values are expressed as peak area values for each compound; values are expressed as peak area values for each compound. d = day, * $p = 0.05$, ND = not detected, NS = not significant.

Table S6: The 26 sensory descriptors used for the evaluation of the 3 pasteurized milk samples (grass (GRS), clover (CLV) and total mixed ration (TMR)) by full descriptive sensory analysis.

Table S1. Microbial results for the raw and pasteurized milk samples (grass (GRS), clover (CLV) and total mixed ration (TMR)). VRB: Violet Red Bile, KAA: Kanamycin Aesculin Azide, MPCA: Milk Plate Count Agar.

			Raw Milk			Pasteurized Milk		
		**Agar Type**	**GRS**	**CLV**	**TMR**	**GRS**	**CLV**	**TMR**
Early lactation		VRB agar	0.0	1.5	0.0	0.0	0.0	0.0
	Bacteria count (Log_{10})	KAA agar	1.0	0.7	1.9	0.7	0.7	1.0
		MPCA	0.0	0.0	0.0	0.0	0.0	0.0
Mid lactation		VRB agar	0.0	0.0	1.4	0.0	0.0	0.0
	Bacteria count (Log_{10})	KAA agar	1.0	1.0	1.7	0.0	0.0	0.0
		MPCA	2.0	0.0	2.3	0.0	0.0	0.0
Late lactation		VRB agar	1.0	1.5	3.8	0.0	0.0	0.0
	Bacteria count (Log_{10})	KAA agar	0.0	1.7	2.4	0.0	0.0	0.0
		MPCA	0.0	0.0	0.0	0.0	0.0	0.0

Table S2. Monthly averages of composition analysis for pasteurized milk samples from cows on different feeding systems; perennial ryegrass (GRS), perennial ryegrass/white clover (CLV) and total mixed ration (TMR) during mid and late lactation.

Component	Diet	Mid Lactation		Late Lactation		
		May	June	September	October	*p*-Value
**Fat %**	GRS	3.4	3.7	2.7	2.8	<0.001
	CLV	3.4	3.4	5.1	4.7	
	TMR	4.2	3.9	2.2	4.4	
**Protein %**	GRS	3.5	3.6	4.0	3.6	<0.001
	CLV	3.6	3.6	4.1	3.8	
	TMR	3.2	3.4	3.9	3.7	
**Lactose %**	GRS	4.8	4.7	4.8	4.3	<0.001
	CLV	5.0	4.7	4.8	4.6	
	TMR	4.8	4.7	5.0	4.7	
**True protein %**	GRS	3.3	3.4	3.8	3.4	<0.001
	CLV	3.4	3.5	3.9	3.6	
	TMR	3.1	3.2	3.7	3.5	
**Casein %**	GRS	2.7	2.7	3.1	2.7	<0.001
	CLV	2.7	2.8	3.2	2.9	
	TMR	2.4	3.2	3.7	2.9	

Table S3. Individual Free Fatty Acid Content mg/kg or ppm (relative standard deviation of the results between replicates as a percent in brackets) for each of the pasteurized (p) milk samples (grass (GRS) grass/clover (CLV) and total mixed ration (TMR)) at day 3, 9 and 14 of refrigerated storage. * p = 0.05, d = day.

Fatty Acid	Grass d 3	Grass/Clover d 3	TMR d 3	Grass d 9	Grass/Clover d 9	Grass d 14	Grass/Clover d 14	TMR d 14	*p*-Value
**C4**	3.0 (41.3)	0.0 (0)	0.0 (0)	3.9 (11.9)	8.5 (11)	0.0 (0)	0.0 (0)	0.0 (0)	*
**C6**	4.4 (4.7)	2.4 (17.1)	2.3 (2.8)	5.3 (2.9)	8.2 (5.2)	6.9 (32.9)	6.4 (14.4)	5.6 (17.3)	*
**C8**	4.2 (4.9)	2.3 (18.5)	2.5 (4.8)	6.0 (0.1)	8.9 (4)	7.6 (32.2)	6.8 (14.3)	6.1 (18.4)	*
**C10**	7.5 (7)	4.3 (15.3)	5.1 (22.2)	11.3 (0.2)	16.7 (3.7)	14.7 (31.7)	12.9 (14.9)	12.0 (17.9)	*
**C12**	8.7 (4.1)	4.9 (15.2)	6.9 (19.5)	12.9 (0.7)	18.7 (2.7)	16.4 (26.2)	13.6 (9.1)	15.0 (16.9)	*
**C14**	15.9 (7.4)	10.2 (16)	10.6 (14.3)	24.3 (0.3)	38.1 (2.8)	33.3 (25.5)	28.1 (6.1)	29.2 (14.8)	*
**C16**	67.4 (5.5)	48.9 (13.8)	51.1 (4.1)	88.2 (2.5)	130.8 (2.6)	122.8 (19.6)	91.6 (2.5)	111.8 (8.5)	*
**C18**	38.7 (6.3)	26.7 (22.3)	30.0 (1.8)	45.5 (3.1)	56.8 (2.7)	58.7 (14.9)	42.6 (3.4)	47.6 (4.6)	*
**C18:1**	27.1 (4.2)	20.1 (36.4)	14.4 (3.1)	63.9 (1)	92.8 (0.5)	99.9 (24.3)	78.4 (10.6)	87.0 (10.2)	*
**C18:2**	3.2 (5.7)	2.8 (55.1)	3.6 (29.9)	5.8 (3.8)	8.2 (12.4)	8.8 (27.9)	8.2 (12.2)	10.1 (24.4)	*
**C18:3**	3.7 (1.3)	1.5 (44.7)	4.1 (20.8)	6.2 (6.7)	7.2 (4.3)	5.0 (22.1)	6.0 (2.4)	2.2 (24.3)	*
**Result Total**	183.7 (4.7)	124.1 (21.1)	130.6 (6.1)	273.3 (1.5)	394.8 (2)	374 (22.1)	294.5 (6.8)	326.7 (10.6)	*

Table S4. Individual Free fatty acid content mg/kg (relative standard deviation of the results between replicates as a percent in brackets) for each of the pasteurized (p) milk samples (grass (GRS), grass/clover (CLV) and total mixed ration (TMR)) at day 3, 9 and 14 of refrigerated storage and the significance between the fatty acids analyzed for each sample (GRS, CLV and TMR). * $p = 0.05$, d = day, NS = not significant.

Fatty Acid	Grass d 3	Grass d 9	Grass d 14	*p*-Value	Grass/Clover d 3	Grass/Clover d 9	Grass/Clover d 14	*p*-Value	TMR d 3	TMR d 14	*p*-Value
**C4**	3.0 (41.3)	3.9 (11.9)	0.0 (0)	*	0.0 (0)	8.5 (11)	0.0 (0)	*	0.0 (0)	0.0 (0)	NS
**C6**	4.4 (4.7)	5.3 (2.9)	6.9 (32.9)	NS	2.4 (17.1)	8.2 (5.2)	6.4 (14.4)	*	2.3 (2.8)	5.6 (17.3)	*
**C8**	4.2 (4.9)	6.0 (0.1)	7.6 (32.2)	NS	2.3 (18.5)	8.9 (4)	6.8 (14.3)	*	2.5 (4.8)	6.1 (18.4)	*
**C10**	7.5 (7)	11.3 (0.2)	14.7 (31.7)	NS	4.3 (15.3)	16.7 (3.7)	12.9 (14.9)	*	5.1 (22.2)	12.0 (17.9)	NS
**C12**	8.7 (4.1)	12.9 (0.7)	16.4 (26.2)	NS	4.9 (15.2)	18.7 (2.7)	13.6 (9.1)	*	6.9 (19.5)	15.0 (16.9)	NS
**C14**	15.9 (7.4)	24.3 (0.3)	33.3 (25.5)	NS	10.2 (16)	38.1 (2.8)	28.1 (6.1)	*	10.6 (14.3)	29.2 (14.8)	*
**C16**	67.4 (5.5)	88.2 (2.5)	122.8 (19.6)	NS	48.9 (13.8)	130.8 (2.6)	91.6 (2.5)	*	51.1 (4.1)	111.8 (8.5)	*
**C18**	38.7 (6.3)	45.5 (3.1)	58.7 (14.9)	NS	26.7 (22.3)	56.8 (2.7)	42.6 (3.4)	*	30.0 (1.8)	47.6 (4.6)	*
**C18:1**	27.1 (4.2)	63.9 (1)	99.9 (24.3)	*	20.1 (36.4)	92.8 (0.5)	78.4 (10.6)	*	14.4 (3.1)	87.0 (10.2)	*
**C18:2**	3.2 (5.7)	5.8 (3.8)	8.8 (27.9)	NS	2.8 (55.1)	8.2 (12.4)	8.2 (12.2)	*	3.6 (29.9)	10.1 (24.4)	NS
**C18:3**	3.7 (1.3)	6.2 (6.7)	5.0 (22.1)	NS	1.5 (44.7)	7.2 (4.3)	6.0 (2.4)	*	4.1 (20.8)	2.2 (24.3)	NS

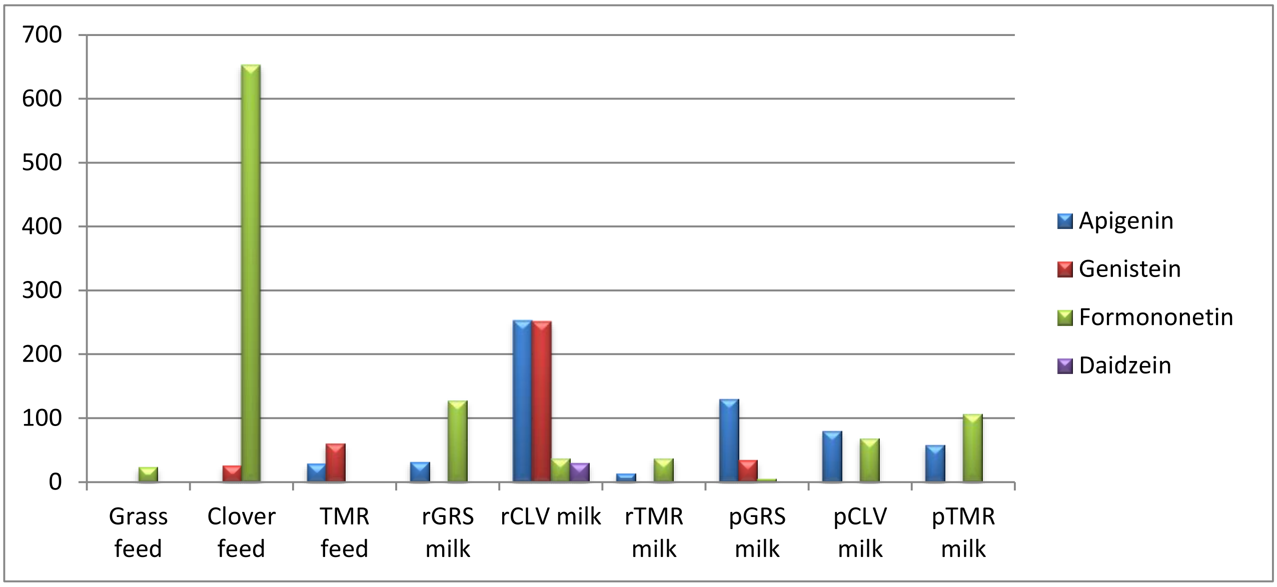

Figure S1. Bar chart showing the levels of important isoflavones in feed samples (grass, clover and total mixed ration (TMR)) and the corresponding raw (r) and pasteurized (p) grass (GRS), clover (CLV) and total mixed ration (TMR) milk samples.

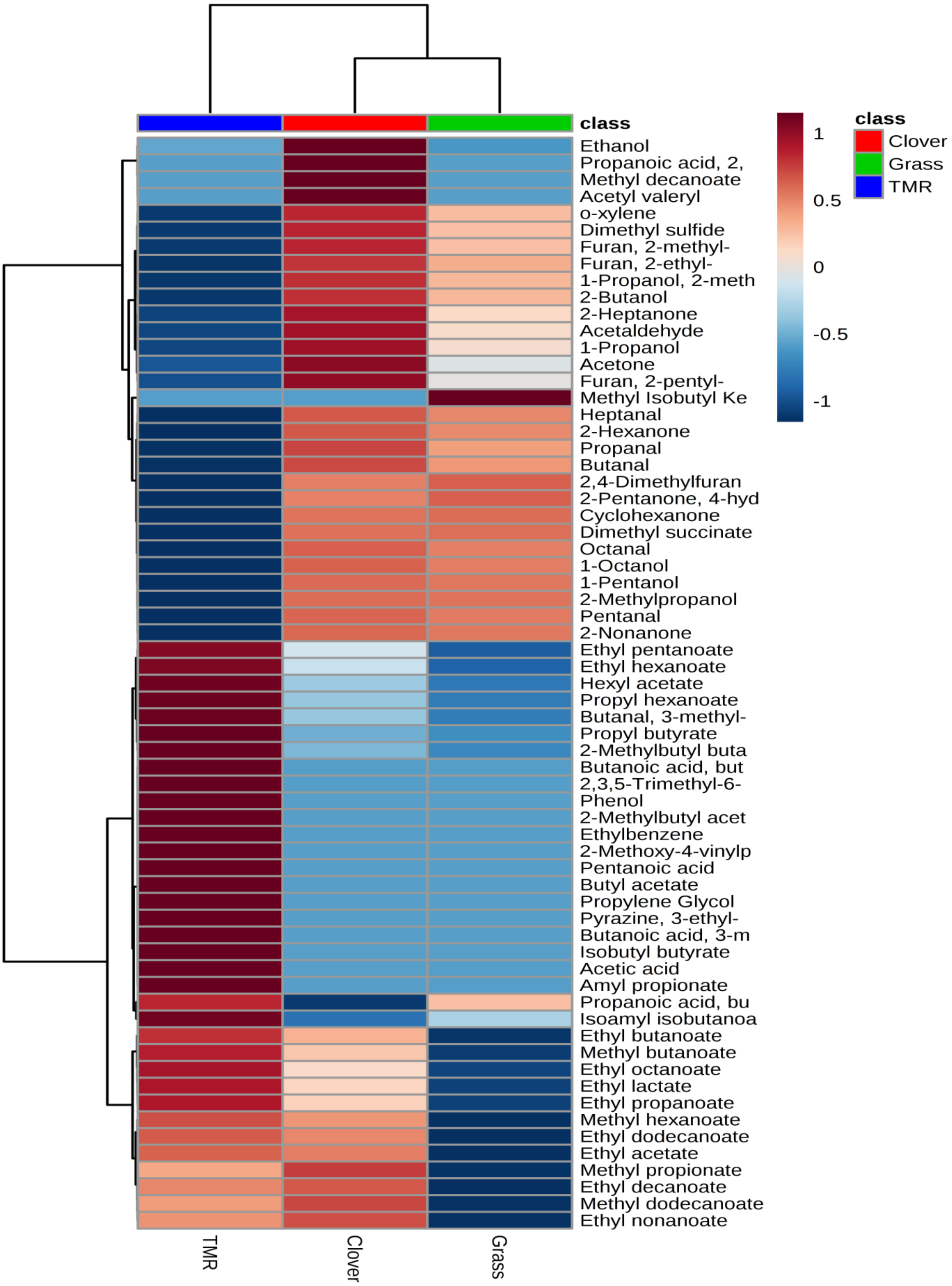

Figure S2. Hierarchal clustering analysis (Heatmap) of the average values for the top 65 volatile organic compounds contributing to the differences between grass, grass/clover and total mixed ration (TMR) feed samples, as determined by headspace solid-phase microextraction gas-chromatography mass spectrometry (HS-SPME GC-MS). Positive and negative correlations between feeding system (grass, grass/clover and TMR) and volatile organic compounds is denoted by +1 (red) and −1 (blue).

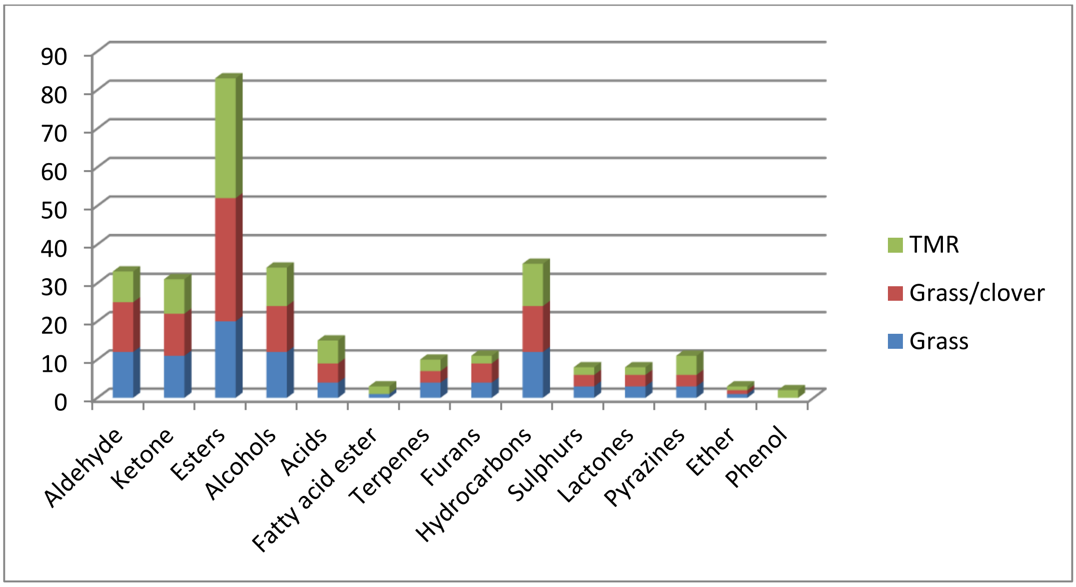

Figure S3. Bar charts showing the percentage of each chemical class (aldehydes, ketones, alcohols, acids, fatty acid esters, terpenes, furans, hydrocarbons, sulphurs, lactones, pyrazines, ether and phenol) identified in each feed type (grass, grass/clover and total mixed ration (TMR). 90, 104 and 94 compounds were identified in grass, grass/clover and TMR feeds, respectively.

Table S5. Relationship between cow diet (grass, grass/clover and total mixed ration (TMR)) and the pasteurized (p) milk volatile compounds identified by headspace solid-phase microextraction gas-chromatography mass spectrometry (HS-SPME GC-MS) at day 3, 9 and 14 of refrigerated storage; values are expressed as peak area values for each compound; values are expressed as peak area values for each compound. d = day, * p = 0.05, ND = not detected, NS = not significant. LRI = Linear retention index.

**Compound**	**CAS No.**	**LRI**	**Grass d 3**	**Grass/Clover d 3**	**TMR d 3**	**Grass d 9**	**Grass/Clover d 9**	**TMR Day 9**	**Grass Day 14**	**Grass/Clover d 14**	**TMR Day 14**	***p*-Value**	***p*-Value (Grass)**	***p*-Value (Grass/Clover)**	***p*-Value (TMR)**
**Aldehyde**															
(*E*)-2-Octenal (or isomer)	2548-87-0	1094	0.00 × 00	0.00 × 00	0.00 × 00	3.80×10^{7}	0.00 × 00	5.05×10^{8}	1.67×10^{8}	0.00 × 00	2.20×10^{7}	*<0.001	NS 0.499	ND	*<0.001
(*Z*)-2-Heptenal (or isomer)	57266-86-1	1012	0.00 × 00	0.00 × 00	0.00 × 00	0.00 × 00	0.00 × 00	0.00 × 00	0.00 × 00	0.00 × 00	2.26×10^{7}	*<0.001	ND	ND	*<0.001
Butanal	123-72-8	627	0.00 × 00	0.00 × 00	0.00 × 00	0.00 × 00	0.00 × 00	0.00 × 00	1.31×10^{6}	0.00 × 00	1.08×10^{7}	*<0.001	NS 0.422	ND	*0.001
3-Methyl-butanal	590-86-3	690	0.00 × 00	0.00 × 00	0.00 × 00	0.00 × 00	0.00 × 00	0.00 × 00	0.00 × 00	0.00 × 00	0.00 × 00	NS 0.469	NS 0.422	ND	ND
Decanal	112-31-2	1250	8.17×10^{6}	4.70×10^{6}	4.45×10^{6}	1.46×10^{7}	2.56×10^{6}	6.46×10^{6}	1.06×10^{7}	4.68×10^{6}	6.58×10^{6}	NS 0.100	NS 0.470	NS 0.211	NS 0.825
Heptanal	111-71-7	941	2.64×10^{8}	2.74×10^{8}	1.33×10^{8}	1.98×10^{8}	1.81×10^{8}	0.00 × 00	6.35×10^{8}	2.52×10^{8}	1.00×10^{9}	*<0.001	*<0.001	* 0.009	*<0.001
Hexanal	66-25-1	838	3.91×10^{8}	3.65×10^{8}	5.35×10^{8}	2.74×10^{8}	2.50×10^{8}	0.00 × 00	6.33×10^{8}	2.91×10^{8}	4.45×10^{9}	*<0.001	*0.018	*0.031	*<0.001
Nonanal	124-19-6	1147	1.49×10^{8}	2.31×10^{8}	1.83×10^{8}	1.28×10^{8}	9.04×10^{7}	4.55×10^{7}	8.62×10^{7}	8.08×10^{7}	1.42×10^{8}	*<0.001	NS 0.120	*0.009	*<0.001
Octanal	124-13-0	1044	4.97×10^{7}	6.24×10^{7}	4.56×10^{7}	4.54×10^{7}	3.49×10^{7}	0.00 × 00	4.38×10^{7}	5.88×10^{7}	1.09×10^{8}	*<0.001	NS 0.867	*0.012	*<0.001
Pentanal	110-62-3	733	7.14×10^{8}	7.90×10^{8}	6.47×10^{7}	5.25×10^{8}	7.04×10^{8}	0.00 × 00	5.87×10^{8}	6.29×10^{8}	2.46×10^{8}	*<0.001	*0.014	*0.025	*0.018
**Ketone**															
2-Butanone	78-93-3	637	6.30×10^{7}	1.04×10^{8}	1.53×10^{8}	7.61×10^{7}	9.97×10^{7}	8.76×10^{7}	7.26×10^{7}	9.84×10^{7}	1.43×10^{8}	*0.001	NS 0.868	NS 0.892	*0.001

2-Heptanone	110-43-0	933	3.73×10^{7}	3.80×10^{7}	3.18×10^{7}	1.40×10^{8}	4.27×10^{7}	7.14×10^{9}	6.77×10^{8}	6.23×10^{7}	5.85×10^{7}	*<0.001	NS 0.416	* 0.011	*<0.001
2-Hexanone	591-78-6	831	1.10×10^{7}	6.07×10^{6}	4.99×10^{6}	1.51×10^{7}	1.15×10^{7}	6.02×10^{7}	2.74×10^{7}	2.49×10^{7}	1.66×10^{7}	*<0.001	NS 0.281	* 0.003	*<0.001
2-Nonanone	821-55-6	1137	0.00 × 00	0.00 × 00	0.00 × 00	8.11×10^{7}	0.00 × 00	2.15×10^{9}	1.97×10^{8}	0.00 × 00	0.00 × 00	*0.001	NS 0.556	ND	*<0.001
2-Octanone	111-13-7	1034	5.44×10^{6}	5.16×10^{6}	3.37×10^{6}	1.27×10^{7}	1.89×10^{7}	3.45×10^{7}	2.69×10^{7}	1.87×10^{7}	1.43×10^{7}	NS 0.071	NS 0.330	NS 0.226	*0.011
2-Pentanone	107-87-9	727	7.97×10^{7}	6.78×10^{7}	6.73×10^{7}	1.06×10^{8}	5.82×10^{7}	7.97×10^{8}	1.40×10^{8}	6.48×10^{7}	6.08×10^{7}	*<0.001	NS 0.726	NS 0.235	*<0.001
2-Undecanone	112-12-9	1353	0.00 × 00	0.00 × 00	0.00 × 00	8.09×10^{6}	0.00 × 00	1.50×10^{8}	1.86×10^{7}	0.00 × 00	0.00 × 00	*0.001	NS 0.516	ND	*<0.001
3-Hexen-2-one	763-93-9	839	1.20×10^{7}	1.70×10^{6}	0.00 × 00	5.93×10^{6}	9.68×10^{6}	1.05×10^{7}	3.80×10^{6}	0.00 × 00	0.00 × 00	NS 0.065	NS 0.244	NS 0.129	NS 0.111
3,5-(*E*,*E*)-Octadien-2-one (or isomer)	30086-02-3	1130	0.00 × 00	0.00 × 00	0.00 × 00	0.00 × 00	0.00 × 00	0.00 × 00	1.39×10^{7}	0.00 × 00	2.86×10^{7}	*<0.001	*0.002	ND	* 0.001
4-Methyl-3-pentene-2-one (tentative)	141-79-7	839	0.00 × 00	0.00 × 00	0.00 × 00	1.03×10^{7}	1.37×10^{7}	9.34×10^{6}	1.65×10^{7}	1.79×10^{7}	0.00 × 00	*0.001	*0.025	*<0.001	NS 0.211
4,6-Dimethyl-2-heptanone	19549-80-5	-	7.33×10^{6}	9.45×10^{6}	8.92×10^{6}	4.66×10^{6}	3.16×10^{6}	0.00 × 00	9.63×10^{6}	1.22×10^{7}	1.91×10^{7}	NS 0.187	NS 0.766	NS 0.071	NS 0.117
5-Hepten-2-one (tentative)	6714-00-7	921	0.00 × 00	0.00 × 00	0.00 × 00	0.00 × 00	0.00 × 00	7.28×10^{7}	0.00 × 00	0.00 × 00	0.00 × 00	*<0.001	ND	NS	*<0.001
Acetone	67-64-1	532	1.23×10^{9}	9.62×10^{8}	1.20×10^{9}	1.24×10^{9}	5.67×10^{8}	7.51×10^{8}	1.15×10^{9}	6.00×10^{8}	1.20×10^{9}	*0.041	NS 0.959	NS 0.218	*<0.001
Acetophenone	98-86-2	1030	3.90×10^{6}	2.18×10^{6}	3.56×10^{6}	4.80×10^{6}	0.00 × 00	2.70×10^{6}	1.57×10^{6}	5.82×10^{5}	8.14×10^{5}	*0.048	NS 0.260	NS 0.170	NS 0.224
Cyclohexanone	108-94-1	956	8.34×10^{5}	8.05×10^{6}	5.36×10^{6}	1.74×10^{6}	9.14×10^{5}	0.00 × 00	0.00 × 00	1.93×10^{6}	0.00 × 00	NS 0.338	NS 0.574	NS 0.054	NS 0.117
Acetyl valeryl (2,3-heptanedione)	96-04-8	875	3.25×10^{6}	1.46×10^{6}	3.68×10^{6}	5.53×10^{6}	2.14×10^{6}	7.10×10^{6}	2.38×10^{6}	6.73×10^{6}	1.80×10^{6}	NS 0.680	NS 0.701	NS 0.364	NS 0.347

Methyl Isobutyl Ketone	108-10-1	780	3.08×10^{8}	1.84×10^{8}	2.41×10^{8}	3.12×10^{8}	1.67×10^{8}	1.80×10^{8}	3.25×10^{8}	1.70×10^{8}	2.45×10^{8}	*0.045	NS 0.982	0.512	*0.027
**Ester**															
Ethyl heptanoate	106-30-9	1120	0.00 × 00	0.00 × 00	0.00 × 00	0.00 × 00	0.00 × 00	3.77×10^{6}	8.69×10^{6}	0.00 × 00	0.00 × 00	NS 0.421	NS 0.422	ND	*<0.001
Ethyl (*Z*)-2-butenoate	6776-19-8	875	0.00 × 00	0.00 × 00	0.00 × 00	0.00 × 00	0.00 × 00	3.01×10^{7}	1.16×10^{8}	0.00 × 00	0.00 × 00	NS 0.468	NS 0.422	ND	*<0.001
Ethyl acetate	141-78-6	639	0.00 × 00	0.00 × 00	0.00 × 00	7.91×10^{6}	0.00 × 00	6.48×10^{7}	9.66×10^{7}	0.00 × 00	0.00 × 00	NS 0.334	NS 0.449	ND	*<0.001
Ethyl decanoate	110-38-3	1419	0.00 × 00	0.00 × 00	0.00 × 00	0.00 × 00	0.00 × 00	0.00 × 00	2.24×10^{8}	0.00 × 00	0.00 × 00	NS 0.051	NS 0.163	ND	ND
Ethyl hexanoate	123-66-0	1021	0.00 × 00	0.00 × 00	0.00 × 00	1.34×10^{8}	0.00 × 00	3.79×10^{9}	2.37×10^{9}	0.00 × 00	0.00 × 00	0.021	NS 0.436	ND	*<0.001
Ethyl octanoate	106-32-1	1220	0.00 × 00	0.00 × 00	0.00 × 00	0.00 × 00	0.00 × 00	3.01×10^{7}	2.65×10^{8}	0.00 × 00	0.00 × 00	NS 0.445	NS 0.405	ND	*0.002
Ethyl pentanoate	539-82-2	923	0.00 × 00	0.00 × 00	0.00 × 00	0.00 × 00	0.00 × 00	3.07×10^{7}	2.13×10^{7}	0.00 × 00	0.00 × 00	*0.045	NS 0.422	ND	*<0.001
Ethyl propanoate	105-37-3	735	0.00 × 00	0.00 × 00	0.00 × 00	0.00 × 00	0.00 × 00	1.65×10^{7}	5.24×10^{6}	0.00 × 00	0.00 × 00	*0.039	NS 0.422	ND	NS 0.095
Methyl butanoate	105-54-4	747	0.00 × 00	0.00 × 00	0.00 × 00	0.00 × 00	0.00 × 00	2.16×10^{7}	0.00 × 00	0.00 × 00	0.00 × 00	*<0.001	ND	ND	*<0.001
Methyl hexanoate	123-66-0	949	0.00 × 00	0.00 × 00	0.00 × 00	6.18×10^{5}	0.00 × 00	2.74×10^{7}	6.60×10^{5}	0.00 × 00	0.00 × 00	*<0.001	NS 0.629	ND	*0.004
Methyl methacrylate	80-62-6	736	5.44×10^{6}	9.65×10^{6}	6.33×10^{6}	0.00 × 00	0.00 × 00	0.00 × 00	0.00 × 00	0.00 × 00	0.00 × 00	*0.007	NS 0.087	NS 0.079	*<0.001
**Alcohol**															
2-Methyl-1-butanol	137-32-6	715	5.50×10^{7}	8.54×10^{7}	8.58×10^{7}	5.30×10^{7}	5.28×10^{7}	1.09×10^{8}	4.92×10^{7}	4.69×10^{7}	3.56×10^{7}	*<0.001	NS 0.851	*0.002	*0.003
3-Methyl-1-butanol	123-51-3	765	7.22×10^{7}	1.92×10^{7}	2.30×10^{8}	2.13×10^{8}	1.22×10^{8}	3.05×10^{8}	7.19×10^{8}	1.32×10^{8}	1.81×10^{8}	NS 0.321	NS 0.356	NS 0.323	NS 0.404

3-Dimethyl-2-butanol (tentative)	594-60-5	773	2.46×10^6	2.61×10^6	0.00 × 00	0.00 × 00	0.00 × 00	0.00 × 00	4.55×10^6	2.19×10^6	2.83×10^6	NS 0.054	NS 0.118	NS 0.959	*<0.001
Ethanol	64-17-5	505	0.00 × 00	0.00 × 00	0.00 × 00	0.00 × 00	0.00 × 00	7.39×10^7	7.00×10^8	0.00 × 00	0.00 × 00	NS 0.481	NS 0.422	NS	NS 0.082
1-Hexanol	111-27-3	894	1.63×10^6	7.06×10^5	0.00 × 00	0.00 × 00	0.00 × 00	0.00 × 00	0.00 × 00	0.00 × 00	0.00 × 00	*0.001	*<0.001	NS 0.422	ND
2-Ethyl-1-hexanol	104-76-7	1075	4.60×10^7	5.01×10^7	3.65×10^7	0.00 × 00	0.00 × 00	0.00 × 00	0.00 × 00	0.00 × 00	0.00 × 00	*<0.001	*<0.001	*0.001	*<0.001
1-Octanol	111-87-5	1116	0.00 × 00	0.00 × 00	2.56×10^6	0.00 × 00	0.00 × 00	0.00 × 00	0.00 × 00	0.00 × 00	2.20×10^7	*<0.001	ND	ND	*<0.001
1-Pentanol	71-41-0	794	1.11×10^9	1.20×10^9	1.01×10^8	6.45×10^8	7.05×10^8	3.48×10^7	4.18×10^8	3.05×10^8	4.67×10^7	*<0.001	*<0.001	*<0.001	*0.040
Isopropyl Alcohol	67-63-0	451	0.00 × 00	0.00 × 00	0.00 × 00	3.31×10^6	0.00 × 00	3.93×10^7	2.58×10^7	0.00 × 00	0.00 × 00	NS 0.147	*<0.001	ND	NS 0.083
**Acid**															
Propanoic acid, 2-methyl-, 3-hydroxy-2,2,4-trimethylpentyl ester	77-68-9	1460	3.82×10^7	1.38×10^7	0.00 × 00	6.86×10^6	5.48×10^6	0.00 × 00	3.74×10^6	2.31×10^6	0.00 × 00	*0.023	NS 0.121	NS 0.340	ND
**Terpene**															
3-Carene	13466-78-9	1035	0.00 × 00	0.00 × 00	0.00 × 00	5.14×10^6	0.00 × 00	0.00 × 00	0.00 × 00	0.00 × 00	0.00 × 00	NS 0.192	NS 0.276	NS	ND
α-Pinene	80-56-8	953	1.01×10^7	8.21×10^6	3.97×10^6	8.04×10^7	2.80×10^7	3.25×10^7	1.56×10^7	7.54×10^6	3.19×10^6	*<0.001	*0.023	*0.004	*0.020
Cumene	98-82-8	990	5.37×10^5	2.26×10^6	1.07×10^6	3.59×10^6	2.03×10^5	9.07×10^6	5.11×10^6	3.59×10^6	1.31×10^6	*<0.001	NS 0.093	NS 0.130	*0.002
D-Limonene	5989-27-5	1055	2.15×10^7	1.34×10^7	1.08×10^7	6.08×10^6	2.63×10^6	0.00 × 00	0.00 × 00	0.00 × 00	0.00 × 00	*0.001	*0.003	NS 0.148	*0.001
Mesitylene	108-67-8	1028	8.50×10^7	5.33×10^7	5.03×10^7	5.68×10^7	2.55×10^7	5.06×10^7	5.13×10^7	3.32×10^7	3.18×10^7	NS 0.151	NS 0.522	*0.025	NS 0.172

trans-β-Ocimene (or isomer)	3779-61-1	1035	0.00 × 00	0.00 × 00	1.08×10^{7}	4.38×10^{6}	0.00 × 00	0.00 × 00	0.00 × 00	0.00 × 00	0.00 × 00	*0.002	NS 0.422	ND	*0.001
**Furan**															
2,4-Dimethylfuran	3710-43-8	732	1.38×10^{7}	1.26×10^{7}	6.20×10^{6}	1.27×10^{7}	4.19×10^{6}	9.01×10^{6}	9.23×10^{6}	1.22×10^{7}	1.54×10^{6}	NS 0.265	NS 0.820	NS 0.183	*0.020
2,5-Dimethylfuran	625-86-5	734	1.55×10^{7}	1.03×10^{7}	3.76×10^{6}	1.42×10^{7}	4.19×10^{6}	9.01×10^{6}	1.96×10^{8}	1.17×10^{8}	7.36×10^{7}	NS 0.452	NS 0.648	NS 0.618	*0.049
2-Ethylfuran	3208-16-0	717	0.00 × 00	0.00 × 00	0.00 × 00	0.00 × 00	0.00 × 00	0.00 × 00	7.87×10^{6}	1.12×10^{7}	1.54×10^{6}	NS 0.114	NS 0.326	NS 0.422	NS 0.124
**Hydrocarbon**															
2,4-Di-tert-butylphenol	96-76-4	1595	0.00 × 00	0.00 × 00	0.00 × 00	1.30×10^{7}	1.81×10^{6}	9.30×10^{6}	3.94×10^{6}	0.00 × 00	0.00 × 00	*0.021	NS 0.108	NS 0.422	NS 0.089
2,6-Bis(1,1-dimethylethyl)-4-(1-oxopropyl)phenol (tentative)	14035-34-8	1684	2.29×10^{7}	1.12×10^{7}	8.54×10^{6}	2.82×10^{7}	1.49×10^{7}	2.18×10^{7}	1.81×10^{7}	2.89×10^{7}	2.55×10^{7}	NS 0.463	NS 0.801	*0.048	NS 0.163
2,4-Dimethyl-benzaldehyde	15764-16-6	1305	3.50×10^{6}	5.16×10^{6}	3.50×10^{6}	0.00 × 00	0.00 × 00	0.00 × 00	0.00 × 00	0.00 × 00	0.00 × 00	*0.001	*0.008	*0.025	*0.048
Benzene	71-43-2	684	7.00×10^{6}	5.78×10^{6}	4.10×10^{6}	4.71×10^{6}	2.68×10^{6}	6.17×10^{6}	2.55×10^{6}	2.32×10^{6}	4.06×10^{6}	NS 0.542	NS 0.154	NS 0.331	NS 0.756
1,2,3-Trimethyl-benzene	526-73-8	1028	8.50×10^{7}	5.33×10^{7}	5.03×10^{7}	5.68×10^{7}	2.55×10^{7}	5.06×10^{7}	5.13×10^{7}	3.32×10^{7}	3.18×10^{7}	NS 0.151	NS 0.522	*0.025	NS 0.172
1,3-bis(1,1-dimethylethyl)-benzene	1014-60-4	1284	5.04×10^{8}	6.01×10^{8}	4.70×10^{8}	4.46×10^{8}	3.33×10^{8}	5.35×10^{8}	5.30×10^{8}	4.51×10^{8}	3.09×10^{8}	NS 0.247	NS 0.902	*0.003	*<0.001
Ethylbenzene	100-41-4	897	9.72×10^{7}	7.10×10^{7}	5.62×10^{7}	1.37×10^{8}	6.50×10^{7}	1.71×10^{8}	1.96×10^{8}	1.17×10^{8}	7.36×10^{7}	*0.032	NS 0.427	*0.003	*<0.001
o-Cymene	527-84-4	1055	1.22×10^{6}	4.97×10^{5}	0.00 × 00	3.10×10^{6}	4.71×10^{5}	1.25×10^{6}	0.00 × 00	0.00 × 00	0.00 × 00	*0.016	*0.015	NS 0.629	NS 0.422
o-xylene	95-47-6	897	7.42×10^{7}	5.95×10^{7}	5.60×10^{7}	8.02×10^{7}	3.98×10^{7}	1.08×10^{8}	1.04×10^{8}	5.92×10^{7}	5.09×10^{7}	NS 0.185	NS 0.768	*0.010	*0.010

p-Cresol	106-44-5	1182	0.00 × 00	0.00 × 00	0.00 × 00	3.58×10^{8}	1.06×10^{8}	1.51×10^{8}	5.93×10^{7}	2.03×10^{7}	1.88×10^{7}	*0.013	NS 0.116	*<0.001	*<0.001
p-Xylene	106-42-3	895	9.72×10^{7}	7.10×10^{7}	5.62×10^{7}	1.37×10^{8}	6.50×10^{7}	1.71×10^{8}	1.96×10^{8}	1.17×10^{8}	7.36×10^{7}	*0.032	NS 0.427	* 0.003	*<0.001
Styrene	100-42-5	927	4.87×10^{6}	4.63×10^{6}	0.00 × 00	0.00 × 00	1.12×10^{7}	0.00 × 00	7.15×10^{6}	2.14×10^{6}	5.53×10^{6}	NS 0.105	NS 0.241	NS 0.233	NS 0.090
tert-Butylbenzene	98-06-6	1024	1.39×10^{7}	1.71×10^{7}	7.51×10^{6}	1.17×10^{7}	8.23×10^{6}	1.33×10^{7}	1.17×10^{7}	1.29×10^{7}	6.30×10^{6}	NS 0.324	NS 0.889	NS 0.121	NS 0.287
Toluene	108-88-3	792	2.42×10^{9}	1.30×10^{9}	6.28×10^{7}	2.41×10^{9}	1.23×10^{9}	4.91×10^{7}	2.35×10^{9}	1.15×10^{9}	4.73×10^{7}	*<0.001	NS 0.996	NS 0.281	*0.035
**Phenolic**															
Phenol	108-95-2	1093	0.00 × 00	0.00 × 00	0.00 × 00	7.60×10^{6}	1.23×10^{6}	0.00 × 00	4.96×10^{6}	1.05×10^{6}	7.23×10^{5}	NS 0.074	NS 0.343	NS 0.626	NS 0.422
**Sulfur**															
Dimethyl sulfide	75-18-3	536	9.61×10^{6}	5.73×10^{6}	2.29×10^{6}	1.40×10^{7}	2.36×10^{6}	2.89×10^{7}	6.23×10^{7}	1.16×10^{6}	0.00 × 00	NS 0.402	NS 0.501	NS 0.409	*<0.001
Dimethyl sulfone	67-71-0	1052	1.77×10^{7}	3.34×10^{6}	0.00 × 00	2.60×10^{7}	9.68×10^{6}	0.00 × 00	1.11×10^{7}	1.34×10^{6}	0.00 × 00	*0.001	NS 0.277	NS 0.200	ND
Methanethiol	74-93-1	459	0.00 × 00	0.00 × 00	0.00 × 00	0.00 × 00	0.00 × 00	9.25×10^{6}	0.00 × 00	0.00 × 00	0.00 × 00	*<0.001	ND	ND	*<0.001
**Ether**															
Ethyl ether	60-29-7	514	8.30×10^{6}	1.01×10^{7}	9.61×10^{6}	7.75×10^{6}	7.17×10^{6}	2.51×10^{6}	5.50×10^{6}	4.57×10^{6}	4.81×10^{6}	NS 0.886	NS 0.865	NS 0.661	NS 0.399
Vinylisopentyl ether	39782-38-2	767	3.91×10^{7}	2.04×10^{8}	2.96×10^{8}	1.84×10^{8}	6.42×10^{7}	1.97×10^{8}	1.21×10^{8}	6.23×10^{7}	5.10×10^{7}	NS 0.111	NS 0.126	NS 0.397	NS 0.094

Table S6. The 26 sensory descriptors applied to the three pasteurized milk samples (grass (GRS), clover (CLV) and total mixed ration (TMR)) by full descriptive sensory analysis.

Section	Descriptor
**Aroma**	
	**1.** Dairy sweet aroma
	**2.** Cooked milk
	**3.** Barnyard aroma
	**4.** Grassy aroma
	**5.** Hay like aroma
	**6.** Malty aroma
**Flavor**	
	**7.** Dairy sweet flavor
	**8.** Cooked milk flavor
	**9.** Dairy fat flavor
	**10.** Malty flavor
	**11.** Creamy flavor
	**12.** Hay like flavor
	**13.** Grassy flavor
	**14.** Dairy sour flavor
	**15.** Off flavor
**Mouth feel**	
	**16.** Viscosity
	**17.** Creaminess
	**18.** Mouth coating
	**19.** Chalkiness
**After effect (AE)**	
	**20.** Astringency
	**21.** Mouth coating AE
	**22.** Dairy sweet AE
	**23.** Cooked milk AE
	**24.** Dairy sour AE
	**25.** Malty AE

26. Barnyard AE
